# Long-read RNA sequencing unveils a novel cryptic exon in *MNAT1* along with its full-length transcript structure in TDP-43 proteinopathy

**DOI:** 10.1038/s42003-025-08463-4

**Published:** 2025-07-16

**Authors:** Yoshihisa Tanaka, Naohiro Sunamura, Rei Kajitani, Marie Ikeguchi, Ryo Kunimoto

**Affiliations:** Research Innovation Planning Department, Research & Development Division, Daiichi Sankyo Co., Ltd., Tokyo, Japan

**Keywords:** Mechanisms of disease, Computational biology and bioinformatics, Amyotrophic lateral sclerosis, Motor neuron disease

## Abstract

Understanding the role of transcript isoforms is essential for elucidating disease mechanisms. TDP-43 regulates RNA splicing, and its dysfunction in neurons is a hallmark of some neurodegenerative diseases, including amyotrophic lateral sclerosis (ALS) and frontotemporal degeneration (FTD). While an association between TDP-43-dependent cryptic exons and disease pathogenesis has been suggested, an approach to investigate how cryptic exons disrupt transcript isoforms has yet to be established. In this study, we developed IsoRefiner, a novel method for identifying full-length transcript structures using long-read RNA-seq. Leveraging this method, we performed long-read RNA-seq, guided by prior short-read RNA-seq, to comprehensively determine the full-length structures of aberrant transcripts due to TDP-43 dysregulation in human iPSC-derived motor neurons. We identified a novel TDP-43-dependent cryptic exon in the *MNAT1* gene, along with its full-length transcript structure. Furthermore, we confirmed the presence of the *MNAT1* cryptic exon in patients with ALS and FTD. Our findings deepen understanding of TDP-43 proteinopathy and advance splicing research.

## Abstract (web summary)

TDP-43 dysfunction leads to aberrant splicing. We developed IsoRefiner to identify full-length transcripts via long-read RNA-seq and found a cryptic exon in the MNAT1 gene in ALS and FTD patients, suggesting its role in TDP-43 proteinopathy.

## Introduction

Splicing is a fundamental process that generates multiple isoforms from a single gene and plays a crucial role in maintaining cellular diversity and homeostasis^1^. The splicing of pre-mRNA is carried out by a large molecular complex known as a spliceosome, which recognizes splice sites at exon-intron junctions^2^. This spliceosome is composed of small nuclear RNAs and various proteins that work together to orchestrate the complex splicing mechanism^2^. Among these components, RNA-binding proteins are particularly important, as they bind to pre-mRNA and influence exon inclusion or exclusion, as well as the use of alternative 5′ and 3′ splice sites^3,4^. Accurate recognition of exons by RNA-binding proteins is a key step in the conversion of pre-mRNAs into mature mRNAs. Disruption of splicing events due to mutations in RNA-binding proteins has been shown to be associated with various diseases, including cancer and neurodegenerative disorders^1,4,5^.

TAR DNA binding protein-43 (TDP-43), encoded by the *TARDBP* gene, is an RNA-binding protein that specifically interacts with UG repeat sequences in RNA, playing an important role in splicing and RNA metabolism^6^. Experiments involving the depletion of TDP-43 in cells have revealed an intriguing phenomenon: the inclusion of an intronic region into some TDP-43 target mRNAs^7,8^. This intronic region is commonly referred to as a *cryptic exon*, defined as an exon that is not present under normal conditions, appears for the first time upon TDP-43 depletion, and is not annotated in any existing reference databases^9^. The binding of TDP-43 to RNA is supposed to suppress the inclusion of cryptic exons into mRNA, thereby preventing the generation of aberrantly spliced mRNA^9^. Accumulating evidence indicates that abnormal aggregation and mislocalization of TDP-43 in the cytoplasm are key features of diseases such as ALS and FTD, which are characterized as TDP-43 proteinopathy^5,10,11^. Recent studies have established a connection between TDP-43 proteinopathy and cryptic exons in the genes *STMN2*^12–14^, *UNC13A*^15,16^ and *HDGFL2*^17^, underscoring the importance of investigating cryptic exons^9^. Given the toxic effects of TDP-43-dependent cryptic exons on cells, elucidation of the full-length transcripts that contain these cryptic exons would potentially deepen our understanding of TDP-43 proteinopathy^17^. However, investigations into the full-length structures of cryptic exon-containing transcripts remain insufficient.

One valuable technology for analyzing splicing is RNA-seq^18^. Indeed, cryptic exons induced by loss of TDP-43 have been identified using short-read RNA-seq^7^, which allows us to investigate splicing events with a large number of reads spanning splice junctions. To this end, numerous bioinformatic tools have been developed to analyze splicing events, significantly advancing the field of splicing research^19–22^. While short-read RNA-seq can accurately capture splicing changes at local level, it struggles to reconstruct full-length transcripts composed of multiple exon-intron structures on a global scale^18^. Efforts have been made to estimate original transcript structures by reassembling short reads^23–26^, but this inherent limitation remains a significant challenge^27^. This issue primarily arises from the fragmentation step during library preparation for short-read RNA-seq, which is technically unavoidable^27^. As a solution to this challenge, long-read sequencing presents a promising technology^28^. Its capability to sequence full-length transcripts could facilitate the identification of structures and the quantification of transcript isoforms, thus addressing the limitation of short reads. The advent of long-read sequencers has driven the development of tools for transcriptomic analysis in recent years^28,29^. The Long-read RNA-Seq Genome Annotation Assessment Project Consortium has evaluated the performance of long-read analysis tools in terms of transcript isoform construction^30^. Unfortunately, the results indicated limited overlap among the tools and low recall rates for isoform construction, with no tool achieving recall greater than 0.5 for manually annotated isoforms. Recall (true positive rate) is the proportion of true transcript structures that the tool can reconstruct. A value below 0.5 indicates that the tool has reconstructed fewer than half of the transcripts that it should detect, which in turn brings a significant negative impact on downstream analyses. Poor performance was also observed in benchmarks using spike-in RNAs, where no tool demonstrated precision exceeding 0.4 for spike-in isoforms not included in the reference data^31^. Precision (positive predictive value) is the proportion of the reconstructed transcript structures that are correct, serving as an indicator of accuracy. While precision and recall often involve a trade-off, both metrics are critical in downstream analyses. However, the studies referenced above indicate some instances where recall and precision are low, underscoring potential concerns regarding overall accuracy. These findings suggest that no single tool currently possesses sufficient performance for accurate isoform analysis. Although long-read technology holds great potential for such analysis, the transcriptome analysis tools are still in the developmental phase and require further refinement.

These considerations motivated us to investigate how improvements in long-read analysis enable the exploration of aberrant splicing induced by the loss of TDP-43 and its impact on transcript structure. Here, we report that *MNAT1* harbors a TDP-43-dependent cryptic exon in neurons, highlighting a previously unrecognized aspect of its splicing regulation. To this end, we developed a method called IsoRefiner to analyze the long-read transcriptome. By leveraging IsoRefiner, we performed both long- and short-read RNA-seq to comprehensively determine the full-length structures of aberrant transcripts resulting from TDP-43 dysregulation in induced pluripotent stem cell (iPSC)-derived motor neurons. Our results revealed a novel TDP-43-dependent cryptic exon in the *MNAT1* gene, for which we identified the full-length transcript structure. Notably, this cryptic exon was detected not only in iPSC-derived motor neurons but also in other neuronal cell contexts and in ALS-FTD patient samples, suggesting the potential involvement of *MNAT1* in TDP-43 proteinopathy.

## Result

### Performance assessment of long-read transcriptome tools and development of the enhanced workflow IsoRefiner

**Fig. 1:**
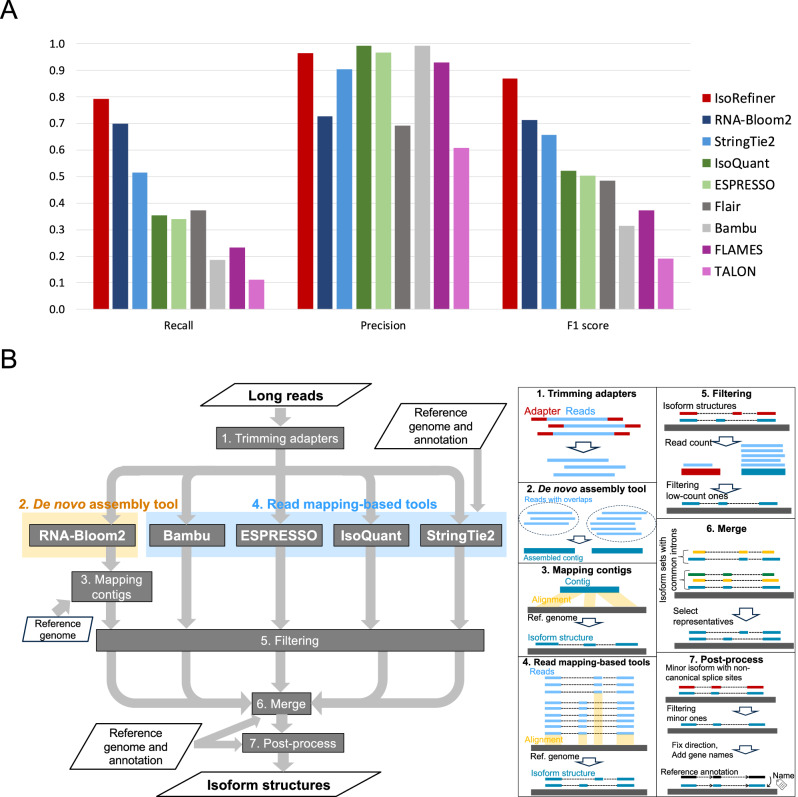
Evaluation of long-read analysis tools and development of IsoRefiner. **A** Performance evaluation of several long-read analysis tools using simulation datasets. Metrics for recall, precision, and F1 score are defined in Method. **B** Schematic overview of our proposed method, IsoRefiner.

Considering that there is still scope to improve the performance of transcriptome analysis tools for long-read sequencing^30^, we first assessed several existing tools using simulation data (5 M reads) to evaluate their ability to accurately construct novel transcript structures (see Materials and Methods for details). In this context, recall refers to the rate of correctly constructed transcript isoforms not found in the input reference annotation with sufficient reads (simulated count ≥10), and precision refers to the rate of correct transcript isoform structures to all predicted ones. In our analysis, no existing tools demonstrated outstanding performance (Fig. 1A). Notably, all mapping-based tools (StringTie2^26^, IsoQuant^32^, ESPRESSO^33^, Flair^34^, Bambu^35^, TALON^36^, and FLAMES^37^ exhibited low recalls ( <0.52). The de novo assembly tool RNA-Bloom2^38^ showed relatively high recall but low precision, achieving the highest F1 score (0.72) among the evaluated tools. To enhance performance, we developed a novel workflow, IsoRefiner, that filters and merges transcript isoform structures from multiple tools (Fig. 1B). First, five tools (Bambu, ESPRESSO, IsoQuant, StringTie2, and RNA-Bloom2) were run independently, allowing flexibility of tool selection based on users’ computational resources. Our tool selection was guided by their performance metrics (Fig. 1A) and associated computational costs (the number of tools). We found that Flair, TALON, and FLAMES all exhibited low F1 scores, with Flair’s particularly low precision, which prompted us to determine that they negatively affect the downstream merged results. Although Bambu also had a low F1 score, it achieved the highest precision among the evaluated tools, leading us to include it in our analysis. Second, isoforms with insufficient supporting reads were filtered out as erroneous. Third, the filtered isoform sets were merged based on matching intron chain (splice sites). Finally, the merged set was refined by considering structural similarities among isoforms, coverage depth, and adherence to the GT-AG splice sites (see Material and Methods for details). Our workflow outperformed other tools, achieving the highest recall (0.79) and F1 score (0.87) without a substantial loss of precision (0.96) (Fig. 1A), indicating the effectiveness of our method.

As a supplementary analysis, to examine whether long reads are more suitable for transcript structure reconstruction than short reads, we generated short-read simulation data using the same simulator and tested multiple tools: StringTie2^26^, Scallop2^39^, and Cufflinks^40^. Considering the typically higher throughput and lower cost associated with short reads, we set the read count to 50 million, which is 10 times more than the long-read data. Despite merging the outputs of these three tools using GffCompare^41^, neither the recall nor the F1 score reached 0.6, demonstrating substantially poorer performance compared to the long read-based results (Supplementary Fig. 1).

### Characterization of iPSC-derived motor neurons and effects of TDP-43 knockdown

**Fig. 2:**
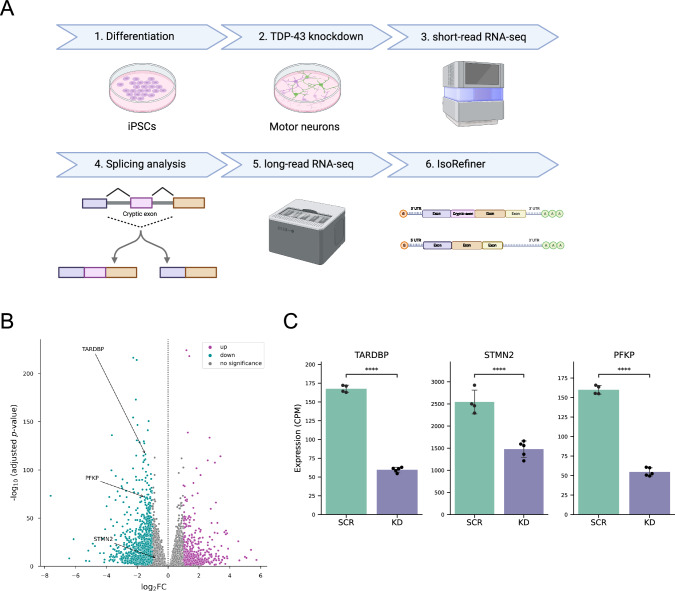
Motor neuron differentiation and TDP-43 knockdown. **A** Illustration of our experimental scheme. **B** Volcano plot from differential gene expression analysis between scramble (SCR, *n* = 4) and TDP-43 knockdown conditions (KD, *n* = 5) in motor neurons using short reads. The fold change (FC) is calculated as KD/SCR. Significant genes are defined using thresholds of adjusted *p*-value < 0.05 and |log_2_FC | ≥1. Upregulated genes are shown in magenta, downregulated genes in cyan, and non-significant genes in gray. **C** Quantification of mRNA expression of *TARDBP*, *STMN2*, and *PFKP* in SCR (*n* = 4) and KD (*n* = 5) motor neurons. CPM, count per million. The column and error bars represent the mean and standard deviation, respectively. ****, adjusted *p*-value < 0.0001 (DESeq2).

TDP-43 pathology is thought to arise from both loss and gain of function mechanism, with the loss of function being strongly associated with splicing alterations observed in the neurodegenerative diseases^11–17,42,43^. In light of the well-established role of TDP-43 in suppressing cryptic exons, we employed a TDP-43 loss-of-function model in motor neurons to investigate transcript structures and demonstrate the applicability of our developed methodology. To this end, we first differentiated human iPSCs into motor neurons^44^ (Fig. 2A). To assess the extent of successful motor neuron differentiation, we performed bulk RNA-seq using short reads for both iPSCs and the differentiated cells. Principal component analysis (PCA) and correlation analysis showed huge differences in transcriptome profiles between these cell types (Supplementary Fig. 2A and 2B). In the differentiated cells, we confirmed the loss of pluripotency markers, including *POU5F1*, *NANOG*, and *SOX2*, along with elevation of neuronal markers such as *ISL1*, *MNX1*, and *MAP2*, at the mRNA expression level (Supplementary Fig. 2C). These findings demonstrated that we successfully generated motor neurons from iPSCs.

Subsequently, we knocked down TDP-43 in these motor neurons using shRNA designed for the *TARDBP* gene (Fig. 2A). To evaluate the effects of TDP-43 knockdown, we performed bulk short-read RNA-seq on samples with scramble shRNA and those with TDP-43 knockdown. PCA indicated that the first principal component accounted for most of the variance in transcriptome profiles between the two conditions (Supplementary Fig. 3A and B). Analysis of differentially expressed genes revealed that this knockdown caused the upregulation of 852 genes and downregulation of 1449 genes (Fig. 2B). The mRNA expression level of *TARDBP* decreased significantly by approximately 70% (Fig. 2C). As markers of TDP-43 loss of function, we also observed a significant decrease in *STMN2* and *PFKP* mRNA expression (Fig. 2C). Additionally, we confirmed the presence of a known cryptic exon in *STMN2* under TDP-43 knockdown (Supplementary Fig. 3C). We next performed differential splicing analysis between scramble and knockdown samples, identifying 1032 novel splicing events with an adjusted *p*-value < 0.05. To determine whether our identified splicing events encompassed those triggered by TDP-43 loss of function, we cross-referenced our identified events with publicly available two independent RNA-seq datasets, specifically those generated by knocking down *TARDBP* in iPSC-derived motor neurons^12^ and iPSC-derived cortical-like neurons^15^. As a result, the known splicing events in *STMN2*, *PFKP*, *ELAVL3*, and *HDGFL2* were also confirmed in our model (Supplementary Fig. 3D), which is largely consistent with previous studies on TDP-43 knockdown^12,13,15^. Taken together, these findings confirm that our TDP-43 knockdown model captures key aspects of TDP-43 loss of function in iPSC-derived motor neurons.

### Screening for cryptic exons in motor neurons following TDP-43 knockdown using short-read RNA-seq

**Fig. 3:**
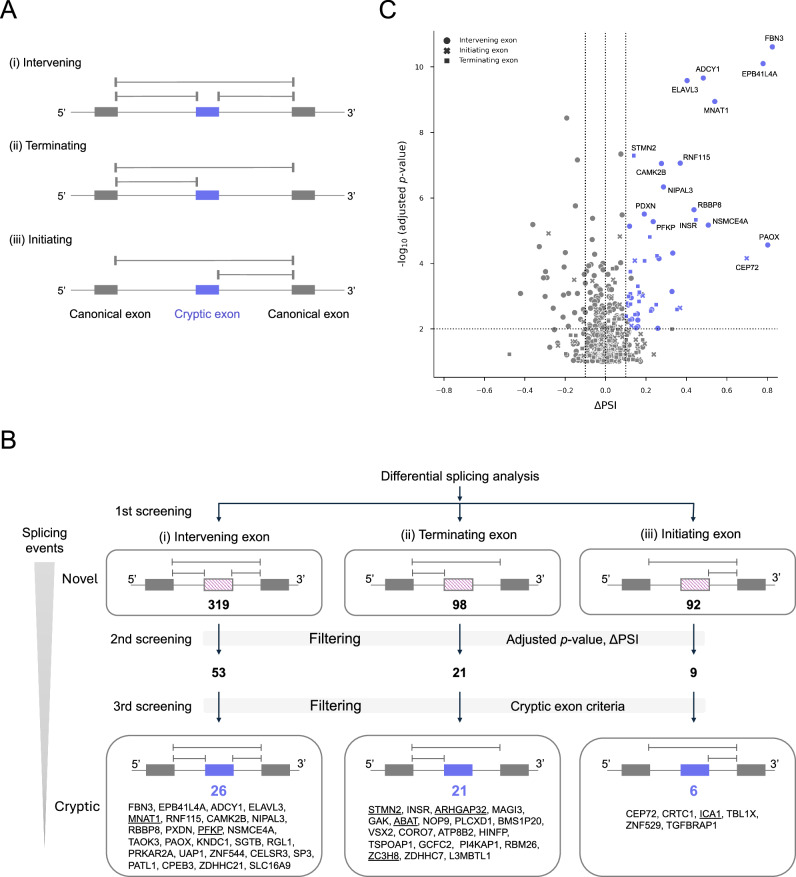
Screening for cryptic exons using short-read RNA-seq. **A** Classification of three patterns of cryptic exons. **B** Screening flow for identifying cryptic exons according to the three patterns using short reads. The underlined genes were ultimately confirmed by long reads (Fig. 4 and Supplementary Fig. 6). **C** Volcano plot from the screening for cryptic exons, showing a total of 509 novel splicing events, including inclusion and exclusion, detected through differential splicing analysis between SCR (*n* = 4) and KD (*n* = 5) conditions in motor neurons. Filtering thresholds for the splicing events are adjusted *p*-value < 0.01 and |ΔPSI | ≥ 0.1, indicated by the dotted lines (black). The final identified significant cryptic exons total 53 (blue), while the remaining unidentified exons number 456 (gray). Circle, cross, and square represent intervening exon, initiating exon, and terminating exon, respectively.

Since the loss of TDP-43 leads to aberrant splicing, we initially screened for cryptic exons using short-read RNA-seq (Fig. 2A). To examine how cryptic exons influence splicing during the transcription process, we categorized our target cryptic exons predicted by short reads into three patterns according to the alignment of short reads spanning splice junctions (Fig. 3A): (i) Pattern 1 refers to an intervening exon, which is conventionally classified as a type of cassette exon where the middle exon among three adjacent exons corresponds to the cryptic exon, and transcription is predicted to continue after the inclusion of the cryptic exon in mRNA. (ii) Pattern 2 refers to a terminating exon, where transcription is predicted to terminate upon the inclusion of the cryptic exon in mRNA. (iii) Pattern 3 refers to an initiating exon, where the inclusion of the cryptic exon is predicted to initiate transcription from that exon (see Materials and Methods for details). In our initial screening, we classified novel splicing events detected by differential splicing analysis between scramble and knockdown conditions with an adjusted *p*-value < 0.1, regardless of whether they involved cryptic exons, into the three exon patterns, identifying 319, 98, and 92 novel splicing events for each pattern, respectively (Fig. 3B). To enhance the confidence in detected novel splicing events, the second screening was applied with more stringent filtering thresholds of adjusted *p*-value < 0.01 and |ΔPSI | ≥ 0.1, resulting in 53, 21, and 9 statistically significant novel splicing events for each pattern (Fig. 3B). In the final screening, we applied our defined criteria for cryptic exons, finally allocating 26, 21, and 6 cryptic exons for each pattern (Fig. 3C). Notably, *STMN2* cryptic exon was classified as a terminating exon, consistent with previous studies reporting that *STMN2* transcription halts at the region of the cryptic exon due to the emergence of a premature termination codon (PTC) and a premature polyadenylation signal^12,13^. In contrast, previously reported cryptic exons in *PFKP* and *ELAVL3*^12^ were classified as intervening exons, suggesting that transcription continues after the inclusion of these cryptic exons.

### Determining structures of cryptic exon-containing transcripts using long-read RNA-seq with IsoRefiner

Although short-read RNA-seq provides clues about splicing events at a local level with high resolution, it cannot clarify the complete structure of transcripts, including the detected splicing events. Therefore, to determine the full-length structure of cryptic exon-containing transcripts, we performed long-read RNA-seq using the Nanopore platform on the same samples as analyzed by short-read RNA-seq (Fig. 2A). Since we employed a polyA-selected method for library preparation, long-read sequencing typically captures the transcript structure of post-spliced mRNA. The mean number of raw reads obtained from all nine samples was 15 million (Supplementary Fig. 4A). The distribution of read lengths exhibited a similar pattern across all samples, with a peak around 900 bp (Supplementary Fig. 4B). Quality checks of the long reads were performed using PCA and correlation analysis at both gene and transcript count levels. These analyses showed distinct transcriptome profiles between scramble and knockdown conditions while maintaining a high correlation among biological replicates, consistent with the short-read data (Supplementary Fig. 5A–D). These read statistics ensured the validity and reproducibility of our long-read sequencing.

**Fig. 4:**
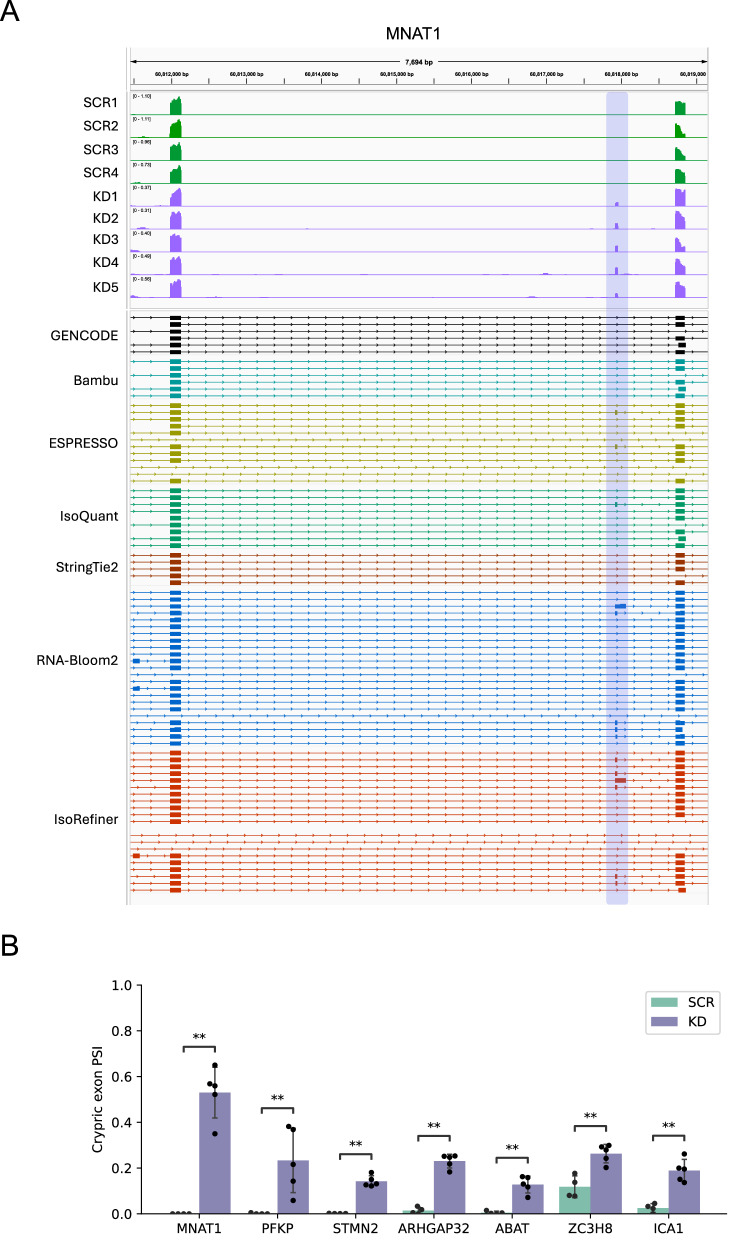
Construction of transcript structures at the *MNAT1* cryptic exon using short- and long-read RNA-seq. **A** Read coverage tracks for SCR (*n* = 4, green) and KD (*n* = 5, purple) motor neurons, generated using short reads. The Y-axis represents CPM. The lower panel shows the transcript structures constructed by each long-read transcriptome analysis tool. The light blue region indicates the position of the cryptic exon. **B** Quantification of cryptic exon inclusion levels (PSI) for the seven targets in SCR (*n* = 4) and KD (*n* = 5) motor neurons. **, adjusted *p*-value < 0.01 (one-sided Mann–Whitney test).

Next, we examined whether long-read RNA-seq was able to identify transcript structures at the positions of cryptic exons detected by short-read sequencing. Given the uncertainty in determining transcript structures when the number of long reads supporting a target splicing event is insufficient, we filtered the total of 53 allocated cryptic exons based on the criterion that the mean of counts per million (CPM) calculated from long reads across all nine samples for the corresponding gene was greater than 5. This filtering resulted in the following final candidates for long reads: (i) *PFKP*, *MNAT1*, *ELAVL3*, *RNF115*, *CAMK2B*, *TAOK3*, *PRKAR2A*, *UAP1*, *CELSR3*; (ii) *STMN2*, *ARHGAP32*, *ABAT*, *ZC3H8*, *VSX2*, *HINFP*, *RBM26*; and (iii) *ICA1*. By using the IsoRefiner workflow, we subsequently performed an integrated visualization analysis that combined short and long reads, allowing us to successfully construct the transcript structures at the sites of cryptic exons predicted by short reads for 7 out of these 17 candidates (Fig. 3B). These candidates included (i) *PFKP*, *MNAT1*; (ii) *STMN2*, *ARHGAP32*, *ABAT*, *ZC3H8*; and (iii) *ICA1* for each respective pattern. (*MNAT1* is shown in Fig. 4A as a representative example, while the other genes are shown in Supplementary Fig. 6.) To investigate whether these seven cryptic exon inclusions occur specifically under TDP-43 knockdown, we quantitatively assessed their inclusion levels (Fig. 4B). Notably, *MNAT1*, *PFKP*, *STMN2* and *ABAT* exhibited pronounced cryptic exon inclusion uniquely in the knockdown condition, which was consistent with the observed read coverage peaks (Fig. 4A and Supplementary Fig. 6).

We also evaluated the performance of other long-read analysis tools in constructing transcript structures for the final 17 candidates. RNA-Bloom2 showed performance comparable to that of IsoRefiner, successfully building transcript structures for 7 of the 17 cryptic exons, while other tools performed less effectively (Table 1). Here, RNA-Bloom2 appears to perform comparably to IsoRefiner; however, its accuracy may be lower than suggested by the simulation-based evaluation (Fig. 1A), potentially including a large number of false-positive structures. To test this, we analyzed the overlap of novel transcripts identified by IsoRefiner and RNA-Bloom2. Using GffCompare^41^, transcripts were considered matching if their splice sites (intron chains) were identical. A Venn diagram showed that RNA-Bloom2 had a substantial number of transcripts unique to RNA-Bloom2’s output (Supplementary Fig. 7A). We further examined the proportion of novel transcripts’ junction sites supported by short reads using SQANTI3^45^. As a result, 37.38% of novel transcripts from IsoRefiner and 14.92% of novel transcripts from RNA-Bloom2 were supported by short reads, suggesting that RNA-Bloom2’s output includes a significant number of false-positive structures.

**Table 1:** Performance comparison of several long-read transcriptome analysis tools in constructing transcript structure for the detected cryptic exons

Gene	Cryptic exon pattern	IsoRefiner	RNA-Bloom2	Bambu	ESPRESSO	IsoQuant	StringTie2
PFKP	Intervening	**+**	**+**				
MNAT1	Intervening	**+**	**+**		**+**	**+**	
STMN2	Terminating	**+**	**+**	**+**	**+**	**+**	**+**
ARHGAP32	Terminating	**+**	**+**				**+**
ABAT	Terminating	**+**	**+**				
ZC3H8	Terminating	**+**	**+**	**+**	**+**	**+**	
ICA1	Initiating	+	+	+	+	**+**	

To underscore the advantages of our combined short- and long-read approach, we independently compared the results from each approach. To this end, we identified significantly altered splicing events (adjusted *p*-value < 0.01 and |ΔPSI | ≥ 0.1) between the TDP-43 knockdown and scramble conditions, subsequently examining the overlaps in genes associated with the events detected by each approach. For short reads, we used our LeafCutter-based approach, while for long reads, we employed IsoRefiner and SUPPA^21^. As a result, only two genes (*MNAT1*, *PFKP*) exhibited overlap, highlighting a substantial difference between the results obtained from each approach (Supplementary Fig. 7B). Notably, the previously reported *STMN2* cryptic exon was detected only in the short read-based result, suggesting that this approach may be more accurate for detecting differential splicing events associated with such cryptic exons. This trend was consistent even when both the short- and long-read datasets were analyzed using the same tool, SUPPA^21^, without novel transcripts (Supplementary Fig. 7C). For transcript structure reconstruction, we tested short read tools (StringTie2^26^, Scallop2^39^, and Cufflinks^40^) to check if the *STMN2* cryptic exon could be reconstructed; however, none of the tools succeeded (Supplementary Fig. 7D). Therefore, we confirmed the validity of our strategy, in which short and long reads were used for differential splicing events and detailed transcript structures, respectively.

### Effect of cryptic exon inclusion on *MNAT1* transcript structure and translation

**Fig. 5:**
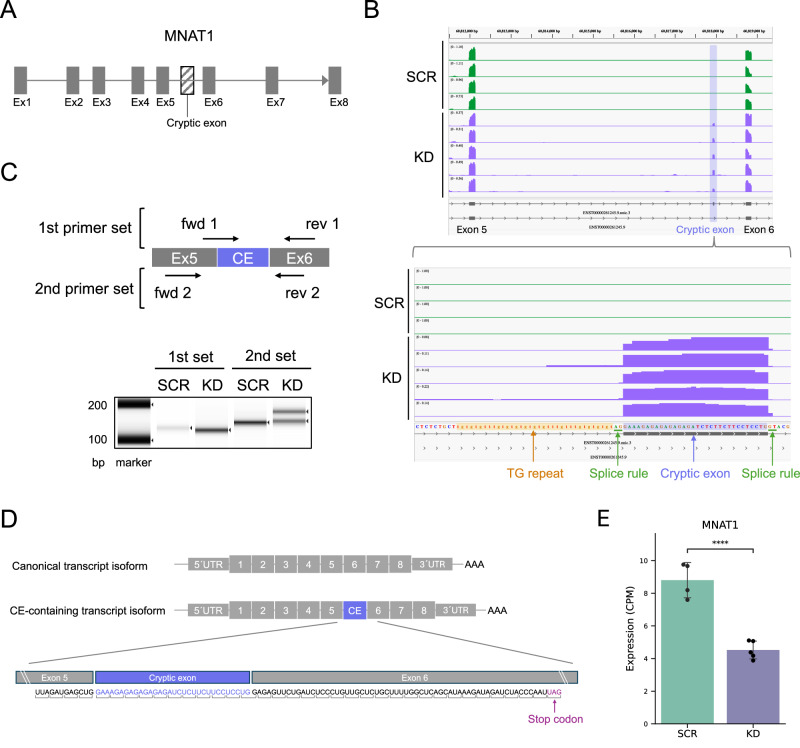
Characterization and functional analysis of the *MNAT1* cryptic exon. **A** Schematic representation of the exon-intron structure of *MNAT1*. **B** Visualization and identification of the *MNAT1* cryptic exon. Read coverage tracks for SCR (*n* = 4, green) and KD (*n* = 5, purple) motor neurons were generated using short reads, with the *MNAT1* transcript structures determined by long reads. **C** RT-PCR in SCR and KD samples using two sets of primers. Forward (fwd) and reverse (rev) primers shown in the figure correspond to each set. **D** Schematic illustration of transcript isoform structures, including both the canonical and cryptic exon-containing variants. Parentheses denote the three codon reading frames. **E** Quantification of *MNAT1* mRNA expression in SCR (*n* = 4) and KD (*n* = 5) motor neurons. Column and error bars represent the mean and standard deviation, respectively. ****, adjusted *p*-value < 0.0001 (DESeq2).

We aimed to address the following key question: What happens to the transcript structure when a cryptic exon is spliced and included in mRNA? Among the seven cryptic exons confirmed by both short- and long-read sequencing, we focused on the *MNAT1* due to its TDP-43 knockdown specific inclusion, highest inclusion level, and broad PSI dynamic range (Fig. 4A and B). We first determined the full-length structures of both the canonical and the cryptic exon-containing transcripts for *MNAT1*. Our long-read sequencing detected the canonical transcript of *MNAT1* (ENST00000261245.9), which consists of eight exons as annotated in GENCODE (Fig. 5A and Supplementary Fig. 8A). We also identified a transcript containing the cryptic exon. Consistent with the prediction that *MNAT1* cryptic exon acts as an intervening exon (Fig. 3B), transcription continued even after its inclusion, producing the transcript composed of nine exons in total, including the cryptic exon alongside the canonical eight exons (Fig. 5A and Supplementary Fig. 8A). PolyA tails were observed at the end of long reads, supporting the full-length structure of these two transcripts (Supplementary Fig. 8B). These results strongly suggest that, in the cryptic exon-containing transcript, exons 6–8 are indeed spliced and transcribed following the inclusion of the cryptic exon. Additionally, differential transcript expression analysis by quantitating long reads indicated that the mRNA expression level of the cryptic exon-containing transcript significantly increased, while that of the canonical transcript decreased (Supplementary Fig. 8C), quantitatively supporting the existence of the cryptic exon-containing transcript.

To obtain further insight into the transcript structure of *MNAT1*, we characterized the profile of the cryptic exon. Both short- and long-read sequencing revealed that the cryptic exon is located between exon 5 and exon 6, with a size of 32 bases (Fig. 5B). Interestingly, this cryptic exon contains several repeat sequences (Fig. 5B). The introns adjacent to the cryptic exon conform to splice site rules, with the intron preceding the cryptic exon ending in an AG sequence and the intron following it beginning with a GT sequence (Fig. 5B). We also observed a TG repeat sequence, which is a known TDP-43 binding motif, in the intron region just before the cryptic exon, further supporting the notion that the *MNAT1* cryptic exon is TDP-43-dependent (Fig. 5B). To confirm the presence of the cryptic exon experimentally, we performed Reverse Transcription (RT)-PCR using two sets of custom-designed primers. The first primer set included a forward primer spanning the splice junction between exon 5 and the cryptic exon, and a reverse primer located at exon 6, allowing direct detection of the *MNAT1* cryptic exon (Fig. 5C). The second primer set was designed to sandwich the cryptic exon, with forward and reverse primers for exon 5 and exon 6, respectively, which would result in an increase in PCR product size by 32 bases if the cryptic exon is spliced into mRNA (Fig. 5C). We detected a specific band and a band shift at the expected size for each primer set, respectively (Fig. 5C). These results reliably validate the presence of the *MNAT1* cryptic exon in the context of TDP-43 depletion in motor neurons.

We next investigated the impact of inclusion of the cryptic exon in mRNA. The canonical transcript of the *MNAT1* gene is generally translated into 309 amino acids, while bioinformatic prediction of the open reading frame (ORF) for the cryptic exon-containing transcript resulted in only 218 amino acids (Supplementary Fig. 8D), indicating that a PTC may appear prior to the final exon 8. Upon manual verification, we confirmed that the inclusion of the *MNAT1* cryptic exon causes an out-of-frame shift, leading to the introduction of a stop codon at exon 6 position (Fig. 5D). The presence of exon-exon junctions downstream of the PTC suggests that this cryptic exon-containing transcript could be targeted by nonsense-mediated decay (NMD)^46^, an innate surveillance system for aberrant mRNA. To investigate this possibility, we compared gene-level expression of *MNAT1* between scramble and knockdown conditions using short-read RNA-seq, finding that *MNAT1* expression was significantly reduced under the TDP-43 knockdown (Fig. 5E). This data offers evidence that the *MNAT1* cryptic exon-containing transcript is likely sensitive to NMD.

### Broader implications of *MNAT1* cryptic exon across iPSC-derived neurons and ALS-FTD patient frontal cortex

**Fig. 6:**
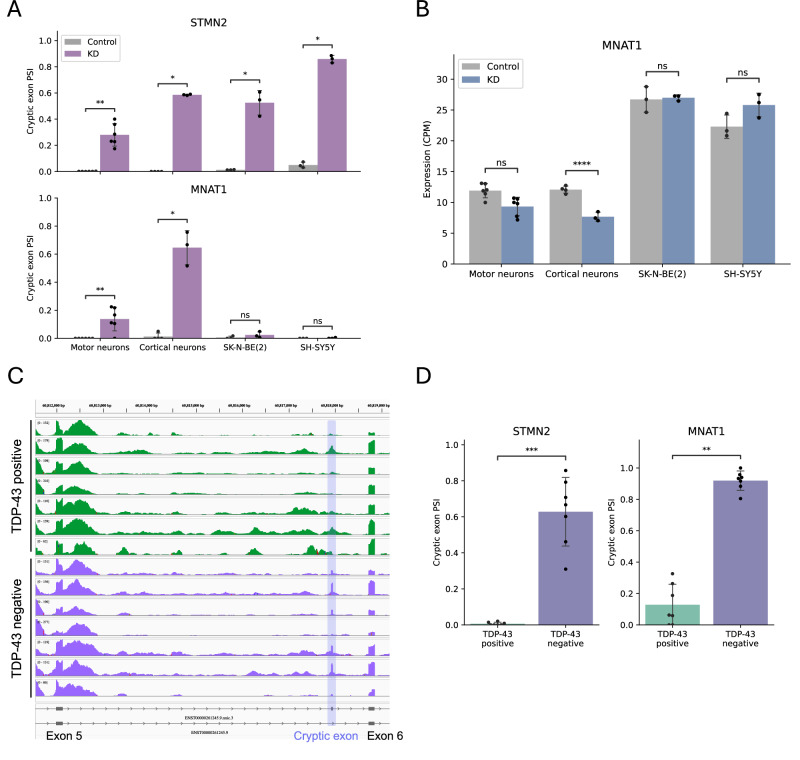
Evaluation of *MNAT1* cryptic exon inclusion in multiple neuronal models and ALS-FTD patient tissues. **A** Quantification of cryptic exon inclusion levels (PSI) under control and TDP-43 knockdown (KD) conditions across four cell types, using previously published datasets: iPSC-derived motor neurons (control and KD each, *n* = 6), iPSC-derived cortical neurons (control, *n* = 4; KD, *n* = 3), and SK-N-BE(2) and SH-SY5Y (control and KD each, *n* = 3) (one-sided Mann–Whitney test). **B** Quantification of *MNAT1* mRNA expression under control and KD conditions in the same cells shown in Fig. 6A (adjusted *p*-values from DESeq2). **C** Visualization of the *MNAT1* cryptic exon in postmortem brain tissue from seven ALS-FTD patients. Read coverage tracks for TDP-43-positive (*n* = 7, green) and -negative (*n* = 7, purple) were generated using short reads. The Y-axis represents CPM. The lower panel shows the *MNAT1* transcript structures determined by long reads. **D** Quantification of cryptic exon inclusion levels (PSI) in TDP-43-positive (*n* = 7) and TDP-43-negative (*n* = 7) nuclei samples, corresponding to Fig. 6C (one-sided Mann–Whitney test). *STMN2* is a positive control. *, adjusted *p*-value < 0.05: **, adjusted *p*-value < 0.01: ***, adjusted *p*-value < 0.001: ns, not significant. Column and error bars represent the mean and standard deviation, respectively.

To investigate the generality of the *MNAT1* cryptic exon, we utilized previously published short-read RNA-seq datasets^12,15^ from in vitro models that induce TDP-43 loss of function in four cell types: iPSC-derived motor neurons, iPSC-derived cortical neurons, and two neuroblastoma cell lines, SK-N-BE(2) and SH-SY5Y. A significant reduction in the expression of both *TARDBP* and *STMN2* upon TDP-43 knockdown supports the validity of the models (Supplementary Fig. 9A). The inclusion level of cryptic exon in *STMN2*, a positive control, was increased across all cell types under the knockdown (Fig. 6A). In contrast, *MNAT1* showed a significant increase in inclusion in motor neurons and cortical neurons, with no such change observed in neuroblastoma cell lines (Fig. 6A). To ask whether the inclusion of cryptic exon affects gene-level expression, we assessed *MNAT1* expression between control and knockdown conditions. In motor neurons and cortical neurons, where cryptic exon inclusion was observed, *MNAT1* expression was reduced compared to the control (Fig. 6B). However, no reduction in expression was observed in neuroblastoma cell lines, where cryptic exon inclusion was not detected (Fig. 6B). These findings suggest that the inclusion of the *MNAT1* cryptic exon leads to decreased *MNAT1* gene expression, consistent with our in-house observations (Fig. 5E).

We finally examined whether the cryptic exon of *MNAT1* also exists in clinical samples. To investigate this, we used a published short-read RNA-seq dataset obtained from the frontal cortex of postmortem brains of seven ALS and FTD patients^47^. In this dataset, TDP-43-positive and -negative nuclei marked with neuronal markers were specifically isolated, with the aim of differentiating pathological cells in the context of TDP-43 proteinopathy^47^. TDP-43 is primarily localized in the nucleus of neurons; however, its abnormal aggregation and mislocalization to the cytoplasm are indicative of a pathological feature in ALS and FTD^5,10,11^. We, therefore, hypothesized that the *MNAT1* cryptic exon might be detected in TDP-43-negative nuclei. By visualizing the aligned reads, we observed coverage peaks at the confirmed position of the *MNAT1* cryptic exon, particularly in the TDP-43-negative nuclei (Fig. 6C). Although the surrounding noise-like reads, likely resulting from the variability of clinical samples, make the peaks less distinct than those observed in the in vitro model, the presence of these peaks certainly corresponds to the cryptic exon in *MNAT1*. To support this observation, we quantified the level of cryptic exon inclusion and found a significant inclusion in the TDP-43-negative nuclei (Fig. 6D). We further examined changes in *MNAT1* expression levels and found no difference between the groups, likely reflecting the nuclear origin of the RNA-seq data (Supplementary Fig. 9B). Collectively, these results demonstrate that the *MNAT1* cryptic exon is present not only in iPSC-derived motor neurons, but also in iPSC-derived cortical neurons and the frontal cortex of ALS-FTD patients, particularly in the context of TDP-43 loss of function.

## Discussion

In this work, we presented a combinatorial approach of short- and long-read RNA-seq to discover a novel cryptic exon in the *MNAT1* gene in a TDP-43-dependent manner. We developed a new tool, IsoRefiner, specifically for analyzing long-read transcriptome data, which enabled us to identify the full-length structure of the cryptic exon-containing transcript in iPSC-derived motor neurons. We further validated the presence of the *MNAT1* cryptic exon not only in iPSC-derived motor and cortical neurons but also in ALS-FTD patients. These findings are highly suggestive of a link between *MNAT1* and TDP-43 proteinopathy. Our study proposes two scenarios regarding how the *MNAT1* cryptic exon may influence cellular function from both transcript and protein perspectives.

In scenario 1, the cryptic exon-containing transcript of *MNAT1* is subject to degradation at the mRNA stage by NMD. In multiple neuronal contexts—including our in-house motor neurons, other groups’ motor neurons, and cortical neurons—*MNAT1* expression levels consistently decrease in response to TDP-43 loss of function when cryptic exons are detected (Fig. 6A and B). In support of this observation, neuroblastoma cell lines lacking cryptic exon inclusion exhibited no change in *MNAT1* expression, effectively serving as a negative control (Fig. 6A and B). These findings suggest that cryptic exon-containing transcripts cause reduced *MNAT1* expression in neurons, hinting at an underlying mechanism likely involving NMD. While we have not shown direct evidence that the cryptic exon-containing transcript is NMD-sensitive, the transcript structural insights obtained from long-read sequencing enable us to hypothesize about the fate of this mRNA. Future experiments using NMD inhibitors would provide further clarity on this interpretation. In scenario 2, the cryptic exon-containing transcript leads to a truncated form of the MAT1 protein, which is encoded by *MNAT1*. MAT1 plays an important role in transcription initiation and DNA repair by forming a tertiary complex with cyclin-dependent kinase (CDK)7 and cyclin H while connecting to the transcription initiation factor IIH (TFIIH) core complex^48,49^. Protein structural study using cryoelectron microscopy unveiled how MAT1 binds to these complexes, specifically indicating that the region of residues 189 to 210 interacts with the XBP unit of the TFIIH complex^50^. Remarkably, this XPB-interacting region aligned perfectly with the area where the reading frame shift induced by the cryptic exon occurs (Supplementary Fig. 10A). Consequently, the amino acid sequence in the truncated MAT1 differs completely from the canonical sequence, indicating a structural alteration in MAT1’s interaction with the TFIIH complex. Furthermore, the C-terminus of MAT1, which interacts with CDK7-cyclin H as an assembly factor for CDK-activating kinase (CAK)^50^, is also compromised in the truncated version due to the early stop codon, resulting in a loss of complex-forming ability (Supplementary Fig. 10A). To structurally evaluate this impairment in complex assembly, we utilized a protein folding artificial intelligence model to perform structural predictions of the ternary complex involving CDK7, cyclin H, and the truncated MAT1. While it is important to note that this analysis is solely based on computational predictions and thus remains speculative, the results showed a disruption of molecular interactions, indicating a potential loss of functionality in the complex (data not shown). Previous studies also showed that MAT1 knockdown leads to decreased phosphorylation of serine 5 on RNA polymerase II (RNApol II), resulting in impairing the transition of RNApol II from transcription initiation to elongation^51^. Upon comprehensive consideration of both our findings and previously accumulated evidence regarding *MNAT1*, inclusion of *MNAT1* cryptic exon would consequently contribute to transcriptomic disruption under either scenario (Supplementary Fig. 10B). However, the pathological significance of the splicing abnormality in *MNAT1* remains unclear and requires further investigation.

Our study surveyed the prevalence of the *MNAT1* cryptic exon under TDP-43 knockdown across various neuronal and neuron-related cell lines, and found that it is detectable in both cortical and motor neurons (Fig. 6A). In contrast, the *STMN2* cryptic exon was observed in all examined cell types, including those with proliferative capacity. These results indicate that the *MNAT1* cryptic exon may be more closely associated with differentiated, non-proliferative cell types that exhibit a more neuron-like transcriptional profile, rather than neuroblastoma cell lines. Our data also uncover the presence of the *MNAT1* cryptic exon in the frontal cortex of patients with ALS-FTD (Fig. 6C and D). Given that the *STMN2* cryptic exon has been proposed as biomarkers for ALS and FTD^13,52^, our findings raise the possibility that the *MNAT1* cryptic exon may similarly serve as a potential biomarker for neurodegenerative disorders associated with TDP-43 proteinopathy, such as ALS and FTD.

We have demonstrated the applicability of IsoRefiner for analyzing long-read transcriptome data using both simulation and actual motor neuron datasets. Our proposed IsoRefiner outperformed existing tools for both datasets. While simulated data may be less complex than real-world data, we observed that current tools exhibited low performance in terms of recall and precision, indicating their immaturity. The ensemble strategy in our IsoRefiner workflow is straightforward and leverages an existing tool (gffcompare)^41^; however, it maintains high precision due to the additional filtering and refinement steps, underscoring the importance of these processes. A limitation of IsoRefiner is its inability to generate structures not present in the results of existing tools, making the selection of appropriate tools critical. Notably, the de novo assembler RNA-Bloom2 outperformed other mapping-based tools in recall for both simulated and actual data. Although RNA-Bloom2 is not widely used and showed low precision compared with the mapping-based tools, our results underscore its potential utility. However, we encountered significant computational challenges with RNA-Bloom2, as it required substantial resources in terms of time and memory. The tool crashed when processing nine samples with an average of 15 million long reads on a machine with 384 GB of memory and 96 cores, prompting us to reduce the input read count by half to successfully execute it. Taking the obtained findings together, this study highlights the importance of not relying on a single tool for long-read RNA-seq analysis, as doing so may lead to incorrect conclusions being drawn. Leveraging a combination of tools could be one option for achieving more robust and reliable results.

Our study has also uncovered some issues associated with long-read RNA-seq data. Since the acquisition of long reads that support transcript structure construction is key to this study, we aimed to maximize read output by sequencing with one flow cell per sample, resulting in an average of 15 million reads per sample. However, this number is still significantly lower than the average of 54 million reads per sample obtained by short-read sequencing. For instance, we encountered cases like *ELAVL3*^12^, where the transcript structure could not be constructed from long reads, even though a cryptic exon was predicted in short reads (Supplementary Fig. 11). One possible explanation for this discrepancy is the insufficient number of long reads to support the structures detected using short reads. Moreover, when counting long reads at the gene level, many genes did not have enough reads to ensure reliable quantification, particularly when applying a cut-off of CPM 5 (Fig. 3B). While it should theoretically be possible to capture novel transcript structures using only long reads, our results indicate that the instability and uncertainty in determining full-length transcript structures pose significant challenges due to the limited number of long reads. Relying solely on long reads may prevent definitive conclusions from being drawn. One potential solution is to increase the number of long reads, but the associated experimental cost remains an issue. Alternatively, combining long- and short-read RNA-seq, as demonstrated in this study, offers a practical approach. The depth of coverage provided by short reads allows precise confirmation of spliced sites, leading to improved and more accurate transcript structures constructed from long reads. Thus, leveraging short reads can effectively address the challenge of long-read RNA-seq and enhance our ability to accurately determine transcript structures.

In summary, we have presented a straightforward approach to investigate how aberrant splicing affects transcript structures and the subsequent impact of these transcripts in cellular functions, made feasible by the integration of both short- and long-read technologies. By determining the full-length structures of transcripts that have undergone aberrant splicing, we can assess the implications for mRNA and explore protein functionalities, including protein structure prediction and potential domain defects. Our analytical approach directly addresses the fundamental principles of transcription and translation outlined in the central dogma of molecular biology. This perspective bridges the hierarchical layers between the transcriptome and the proteome, facilitating our understanding of these complex interactions and advancing our knowledge of molecular biology and disease mechanisms.

## Materials and Methods

### Cell culture

Human iPSCs (771-3 G) were obtained from REPROCELL (Kanagawa, Japan) and maintained in StemFit medium (AK02; Ajinomoto, Tokyo, Japan). We differentiated the iPSC lines into motor neurons using a modified version of a previously reported method^44^. The iPSC lines were dissociated into single cells using StemPro Accutase Cell Dissociation Reagent (Thermo Fisher Scientific, Waltham, MA, USA) and seeded onto 10-cm cell culture dishes coated with iMatrix-511 silk (Nippi, Tokyo, Japan) in StemFit medium supplemented with Y-27632 (10 μM; Cayman Chemical, Ann Arbor, MI, USA) at a density of 2.5 × 10^5^ cells. The initiation of commitment to the motor neuron lineage was defined as day 0, and the cells were maintained in differentiation medium consisting of a 1:1 ratio of DMEM/F-12 (Thermo Fisher Scientific) and Neurobasal medium (Thermo Fisher Scientific), supplemented with 0.5× N2 supplement (Thermo Fisher Scientific), 0.5× B27 supplement (Thermo Fisher Scientific), 0.1 mM ascorbic acid (FUJIFILM Wako Pure Chemical Corporation, Osaka, Japan), and 1× GlutaMax (Thermo Fisher Scientific). From days 1 to 7, the differentiation medium was supplemented with CHIR99021 (3 μM; Wako), DMH1 (2 μM; Wako), and SB431542 (3 μM; Wako). From day 8 to 14, SB431542 (2 μM), DMH1 (2 μM), CHIR99021 (1 μM), retinoic acid (0.1 μM; Wako), and purmorphamine (0.5 μM, Cambridge, MA, USA) were added to the differentiation medium. From day 15, the cells were maintained in differentiation medium containing SB431542 (2 μM), DMH1 (2 μM), CHIR99021 (3 μM), retinoic acid (0.1 μM), purmorphamine (0.5 μM; Wako), and valproic acid (10 μM; Sigma-Aldrich, St. Louis, MO, USA). On day 39, the cells were used for transduction. Knockdown experiments were performed following standard protocols using lentiviral vectors containing either TDP-43 shRNA or scramble shRNA (VectorBuilder Inc., Chicago, IL, USA). On day 46, following 7 days of transduction, the cells were harvested for RNA extraction. Similarly, the non-treated motor neurons used for comparison with iPSCs were harvested on day 46.

### RNA extraction

RNA was extracted from cultured cells using RNeasy Micro Kit (#74004; QIAGEN, Hilden, Germany) or Mini Kit (#74104; QIAGEN) following the QIAshredder (#79656; QIAGEN) and DNase (#79254; QIAGEN) digest protocol with QIAcube (QIAGEN). The quality of the extracted RNA was assessed using TapeStation (Agilent Technologies, Santa Clara, CA, United States), confirming an RNA integrity number of over 9.0 for all samples. The extracted RNA was subsequently used for RT-PCR and short- or long-read RNA sequencing.

### RT-PCR

Reverse transcription was performed using ReverTra Ace qPCR RT Master Mix with gDNA Remover (#FSQ-301; TOYOBO, Osaka, Japan), in accordance with the manufacturer’s protocol. Reverse-transcribed cDNA was subjected to PCR using PrimeSTAR Max DNA Polymerase (#R045; TAKARA, Shiga, Japan) following the manufacturer’s protocol with two sets of custom-designed primers (Thermo Fischer Scientific): The first set are forward 5′-TGAGCTGGAAAGAGAGAGAGAG-3′ (exon 5–cryptic exon) and reverse 5′-AGGTTTGGGTTTCTCAAGTTGC-3′ (exon 6), while the second set was forward 5′-TGGAAGAAGCTTTAGAAGTGGA-3′ (exon 5) and reverse 5′-AGCAACAGGGAGATCAGAACTC-3′ (exon 6). PCR products were analyzed using TapeStation.

### Short-read RNA sequencing

100 ng of total RNA was used as the initial input for polyA selection with NEBNext Poly(A) mRNA Magnetic Isolation Module (#E7490; NEB, Ipswich, MA, United States). The polyA-selected mRNA was then used to prepare a stranded library following the protocol of NEBNext Ultra II Directional RNA Library Prep (#E7765; NEB). Fragmentation of mRNA was performed to make an insert size of approximately 150–300 bp. Dual index adaptors were ligated via PCR using NEBNext Multiplex Oligos for Illumina (#E6440; NEB). Quality assessment of the prepared libraries was performed using TapeStation and Qubit (Thermo Fisher Scientific). In-house sequencing was carried out on the NextSeq1000 system (Illumina, San Diego, CA, United States) with paired-end reads of 100 bp×2, generating an average of approximately 60 million reads per sample across all 17 samples. Raw reads were trimmed and filtered using fastp^53^ (v0.20.1), and the filtered reads were aligned to a human reference genome using STAR^54^ (v2.7.10b). The aligned reads were counted using RSEM^25^ (v1.3.1). The GRCh38 reference genome and reference annotation GENCODE^55^ v42 were used for all analyses in this study.

### Long-read RNA sequencing

200 ng total RNA was used as the initial input for library preparation using PCR-cDNA Sequencing Kit [#SQK-PCS111; Oxford Nanopore Technologies (ONT), Oxford, United Kingdom] following the manufacturer’s protocol. The extension time during the PCR step was set to 60 sec to obtain transcripts of almost 1 kb in size. Quality assessment of the prepared libraries was performed using TapeStation and Qubit. Libraries were sequenced on the GridION system (ONT) using one flow cell (#FLO-MIN106D; ONT) per sample for 72 hr.

### Downstream analysis for short- and long-read RNA-seq

PCA and correlation analysis (using Pearson metrics) were performed on genes filtered using the following criteria across all samples: for short reads, genes with at least four samples (for the TDP-43 knockdown scheme) or three samples (for the motor neuron differentiation scheme) showing CPM ≥ 10; and for long reads, genes with at least four samples across all samples showing CPM ≥ 5 at both gene and transcript levels. Differential gene expression analysis (for short reads) and differential transcript expression analysis (for long reads) were performed between scramble (*n* = 4) and TDP-43 knockdown conditions (*n* = 5) using DESeq2^56^ (v1.42.0) on genes and transcripts, respectively, that had at least four samples across all samples, with raw read counts ≥ 10. The fold change (FC) was denoted as knockdown/scramble. The thresholds for DESeq2 were set to Benjamini-Hochberg adjusted *p*-value < 0.05 and |log_2_FC | ≥ 1. For read visualization, STAR-aligned BAM files were indexed with Samtools^57^ (v1.6), and read coverages were normalized to CPM and exported as a bigwig format file using deepTools^58^ (v3.5.5). The bigwig files were visualized using Integrative Genomics Viewer^59^ (v2.16.2).

### Splicing analysis for identification of cryptic exons using short-read RNA-seq

To perform splicing analysis, LeafCutter^22^ (v0.2.9) was used in accordance with the procedure described in the official manual. BAM files were generated using STAR^54^ with the following specific options: --outSAMstrandField, intronMotif, --twopassMode Basic. These BAM files were converted to junction files using RegTools^60^ (v1.0.0) (“regtools junction extract” command) with the following parameters: -a 8, -m 50, -M 500000, -s RF. Intron clustering was performed for all nine samples (scramble and knockdown) using leafcutter/clustering/leafcutter_cluster_regtools.py with the following parameters: --minclureads 50, --maxintronlen 200000, --mincluratio 0.001. Differential splicing analysis was performed between scramble (*n* = 4) and TDP-43 knockdown conditions (*n* = 5) using leafcutter/script/leafcutter_ds.R with the following parameters: --min_samples_per_intron 3, --min_samples_per_group 3, --min_coverage 20. Output files of LeafCutter were converted into individual files detailing intron and cluster information for downstream analyses using leafcutter/leafviz/prepare_results.R via the RData format. To classify intervening, terminating, and initiating exons, we used an in-house script based on a previous study^61^. Intervening (cassette) exon is defined as follows: (1) the cluster consists of three splice junctions with the same genomic orientation, (2) the central exon is flanked by two splice junctions, (3) of these two splice junctions, the end coordinate of the splice junction closer to 5′ comes before the initial coordinate of the other splice junction, and (4) the longest splice junction that spans the entire cluster is present (Fig. 3A(i)). Terminating and initiating exons are defined as follows: (1) the cluster consists of two splice junctions with the same genomic orientation, (2) the longest splice junction spans the entire cluster, (3) the end coordinate of the other splice junction comes before the end coordinate of the longest splice junction (terminating), or the initial coordinate of the other splice junction comes after the initial coordinate of the longest splice junction (initiating) (Fig. 3A(ii) and (iii)). Inclusion or exclusion is determined by comparing Percent Splice In (PSI) calculated by LeafCutter between two conditions. An exon is classified as novel or annotated based on whether the splice junction is annotated as an exon in GENCODE^55^. A cryptic exon is defined by the following criteria: it is specifically included upon TDP-43 knockdown and is not annotated in GENCODE^55^. To extract clusters with significant changes, we used two indicators calculated by LeafCutter: Benjamini-Hochberg adjusted *p*-value and PSI for each cluster. PSI calculated by LeafCutter (LeafCutterPSI) is assigned for each splice junction. For Fig. 3C, using LeafCutterPSI, we defined PSI at the cluster level for each classified cluster as follows:

${{PSI}}_{{cluster}}=1-{LeafCutterPSI\; of\; longest\; splice\; junction}$. Here, ${{PSI}}_{{cluster}}$ represents the proportion of inclusion reads relative to all reads (inclusions + exclusions) that support the splice junctions within a single cluster. The change in PSI ($\Delta {{PSI}}_{{cluster}}$) is calculated as follows: $\Delta {{PSI}}_{{cluster}}={{PSI}}_{{cluster}}^{s}-{{PSI}}_{{cluster}}^{t}$, where s and t denote TDP-43 knockdown and scramble conditions, respectively.

To quantify the inclusion level of cryptic exon (${{PSI}}_{{cryptic\; exon}}$) within each cluster (as illustrated in Fig. 3A), we calculated PSI using a splice junction-based approach. This approach quantifies the proportion of splice junction reads supporting cryptic exon inclusion relative to all splice junction reads observed within the same cluster as follows:

$${{PSI}}_{{cryptic\; exon}}=\,\frac{{reads\; supporting\; cryptic\; exon\; junctions}}{{total\; reads\; for\; all\; splice\; junctions\; in\; the\; cluster}\,}.$$

Splice junction-spanning reads were identified using RegTools^60^. Splice junctions for PSI calculation are provided in Supplementary Data 1. In this paper, a “cluster” defined by LeafCutter corresponds to a “splicing event.”

### Analysis of public RNA-seq datasets

The public short-read RNA-seq datasets^12,15,47^ were re-analyzed using the same methodology as employed for the in-house analysis. Details of the public datasets are provided in Supplementary Data 2.

### IsoRefiner workflow for long-read RNA-seq analysis

We developed a tool named IsoRefiner to establish a workflow of long-read RNA-seq analysis. This workflow consists of the following steps:

#### (1) Pre-processing of raw reads

FASTQ files of raw reads are pre-processed using Porechop_ABI^62^ (v0.5.0) with the following parameters and options: --ab_initio, --verbosity 1, --threads 48. In this step, adaptors and barcodes in the raw reads are automatically detected and removed.

#### (2) Mapping reads to the reference genome

To generate BAM files for mapping-based tools (StringTie2^26^ (v2.2.1), IsoQuant^32^ (v3.3.1), ESPRESSO^33^ (v1.3.2), and Bambu^35^ (v3.4.0)), pre-processed reads are mapped to the reference genome using Minimap2^63^ (v2.26-r1175) with the following options: -a, -x splice, -ub, -k14. The resulting SAM files are converted to BAM files and sorted using Samtools^57^.

#### (3) Execution of transcript isoform-prediction tools

Bambu^35^, ESPRESSO^33^, IsoQuant^32^, and StringTie2^26^ are executed using the BAM files generated in step (2). RNA-Bloom2^38^ (v2.0.1) is executed with the pre-processed reads (FASTQ files). Detailed methods for each tool are described in the following sections. Note that users can select tools according to their computing resources.

#### (4) Filtering transcript isoform structures

For each GTF file from the transcript isoform prediction tools, isoforms with few reads are filtered as artifacts. First, isoform sequences (FASTA file) are extracted using gffread^41^ (v0.12.7). Next, pre-processed reads are mapped to the isoform sequences using Minimap2^63^ with the following options: -ax map-ont, --secondary=no. Mapped reads are retained if they meet the following criteria: sequence identity ≥90%, unaligned (clipped) length ≤200 bp, and max indel length ≤20 bp. Finally, isoforms are retained if they satisfy the following conditions: coverage (percentage of the region covered by at least one read) ≥95% and mean coverage depth ≥1. Coverages and mean coverage depths are calculated using Samtools^57^ (“samtools coverage” command).

#### (5) Merging transcript isoform structures

To merge transcript isoform sets, the results from step (4) and the reference annotation are input into GffCompare^41^ (v0.12.6) with the option “-p cons.” Among isoforms that have matching intron chains (*i.e*., identical splice sites), the longest isoform is retained as the representative.

#### (6) Post-processing of the merged result

This step removes erroneous transcript isoforms, which are assumed to have a similar structure and fewer reads compared to the closely related isoform. First, the result of the step (5) and the reference annotation are compared using GffCompare^41^ with the default parameters. For each isoform, its strand ( + or −) is adjusted according to the reference isoform with the maximum length of exon overlap. Second, the mean coverage depth of each isoform is calculated using a method similar to that in step (4). Third, for each isoform (*T*), another isoform (*T*_max_ovl_) with the maximum intron-overlap length is identified. This process is performed using Bedtools^64^ (v2.31.0; “bedtools intersect” command) and in-house scripts. Canonical introns are defined as those that starts with “GT” and ends with “AG” (*i.e*., following the GT-AG rule). An isoform (*T*) is excluded if all of the following conditions are satisfied:

(i) The sum of differences of intron positions ≤20 bp.

(ii) *T* consists of a non-canonical intron.

(iii) *T*_max_ovl_ only consists of canonical introns.

(iv) The total length of exons in *T* ≤ the total length of exons in *T*_max_ovl_.

(v) The mean coverage depth of *T* ≤ mean coverage depth of *T*_max_ovl_.

Finally, the resulting transcript isoform set and the reference annotation are compared again using GffCompare^41^ with the option “--strict-match -e 0.” Based on the results from GffCompare^41^ (tmap file), for each merged isoform (name, *s*), the reference isoform with the maximum exon overlap length (name, *s*_ref_) is determined. Then, *s* is updated according to the exact match, *s*_ref_; splice-site (intron-chain) match, *s*_ref_ plus “.nic”; otherwise, *s*_ref_ plus “.nnic.” If no overlap is found, *s* is left unchanged. The GTF file generated by IsoRefiner is provided in Supplementary Data 3.

### Nanopore-read and Illumina short-read simulation

SQANTI-SIM^65^ (v0.2.0) was used to simulate nanopore cDNA reads and Illumina short reads (paired ends) for benchmarking transcript isoform-prediction tools. Subcommands of sqanti-sim.py, namely, “classif,” “sample,” and “sim,” were executed sequentially with the following parameters and options: --ont, --read_type cDNA, --long_count 5000000, --illumina, --short_count 5000000, -k 48, -s 1 (indicating 5 million nanopore reads, 50 million illumina short reads, 48 threads, and a random seed of 1). The input data included the reference genome (“--genome” option) and the reference annotation (“--gtf” option, which contained 252,416 transcript isoforms). This tool generated a reduced genome annotation (GTF file) from which 13,743 transcript isoforms were removed. The reduced GTF file was then used to evaluate the performance of transcript identification tools in identifying novel transcript isoforms.

### Calculation of performance metrics on simulation data

Recall (sensitivity), precision, and F1 score were calculated to evaluate the performance of transcript structure identification on simulation data. We compared each tool’s transcript isoform set to the reference transcript isoform set using GffCompare^41^ with the default parameters, extracting recall and precision at the isoform level from the results of GffCompare^41^. These metrics are based on matches of transcript isoform structures. A match occurs for each pair of tool and reference isoforms, where the lengths are denoted as *T*_*t*_ and *T*_*r*_, respectively, if one of the following conditions is met:

(i) They are multi-exon isoforms, and all intron positions are identical.

(ii) They are single-exon isoforms, meeting either of the following criteria: (overlap length ≥ 0.8×*T*_*t*_ length) or (overlap length ≥ 0.8×*T*_*r*_ length and overlap length ≥ 0.7×*T*_*t*_ length).

The calculations for recall, precision, and F1 score are as follows:

The number of true positives (TP) = the number of tool’s isoforms with matches.

The number of false positives (FP) = the number of tool’s isoforms without matches.

The number of false negatives (FN) = the number of reference isoforms without matches.

The formulas are as follows:

Recall = TP / (TP + FN).

Precision = TP / (TP + FP).

F1 score (harmonic mean of recall and precision) = 2×recall×precision/(recall+precision)

For recall calculation, we used reference isoforms that were absent from the reduced GTF file (removed by SQANTI-SIM^65^ during its generation) and had a number of simulated reads ≥10. All reference isoforms were used for precision calculation. Notably, we intended to measure recall for isoforms with sufficient read support.

### Open reading frame prediction

TransDecoder^66^ (v5.7.1) was used to predict ORFs on transcript isoforms. First, the sequences (FASTA file) of isoforms were extracted using GffRead^41^. Second, these sequences were input into the command “TransDecoder.LongOrfs” with the following parameters and options: -S, --complete_orfs_only, -m 20. Finally, the command “TransDecoder.Predict” was executed with the option “--single_best_only.” For each isoform, the predicted ORF had to meet the following criteria: (i) it was on the plus strand of the isoform (as indicated by the “-S” option), (ii) it contained both start and stop codons (corresponding to the “--complete_orfs_only” option), (iii) its length was ≥ 20 amino acids, and (iv) it was the most strongly supported prediction for that isoform (corresponding to the “--single_best_only” option).

### StringTie2 execution

StringTie2^26^ was executed with the option “-L” (long-read mode) for long reads, and this option was not specified for short reads. The inputs were the mapped reads (BAM files), reference genome, and reference annotation. The mapping tools were Minimap2^63^ and STAR^54^ for long and short reads, respectively.

### IsoQuant execution

IsoQuant^32^ was executed with the mapped nanopore reads (BAM files), reference genome, and reference annotation. The file “isoquant_out/isoquant/isoquant.extended_annotation.gtf” was deemed to be the final output. The commands (options) were as follows:

isoquant.py --threads 96 --reference reference_genome.fa --genedb reference_annotation.gtf --complete_genedb --bam_list input_bam.txt --data_type nanopore --stranded none --output isoquant_out --transcript_quantification unique_only --gene_quantification unique_only --matching_strategy default --splice_correction_strategy default_ont --model_construction_strategy default_ont --no_secondary --check_canonical --count_exons.

### ESPRESSO execution

As the workflow of ESPRESSO^33^, the scripts “ESPRESSO_S.pl,” “ESPRESSO_C.pl,” and “ESPRESSO_Q.pl” were sequentially executed. ESPRESSO_C.pl was applied to each sample. The inputs were the mapped nanopore reads (BAM files), reference genome, and reference annotation. For the TDP-43-related samples, the reads were reduced by half using SeqKit^67^ (v2.4.0; “seqkit sample -p 0.5” command) because of a memory-usage issue. All parameters but the number of threads (S, 48; C, 96; Q, 48) had the default values. The file “espresso_work/samples_N2_R0_updated.gtf” was deemed to be the final output.

### Bambu execution

Bambu^35^ was executed as an R script containing the “bambu” and “writeBambuOutput” functions. The inputs were the mapped nanopore reads (BAM files), reference genome, and reference annotation. All parameters but “ncore=48” of the “bambu” function were default values. The isoforms with read counts of zero were excluded using the in-house script, and the resulting GTF file was deemed to be the final output.

### RNA-Bloom2 execution

A de novo assembler for long-read RNA-seq data, RNA-Bloom2^38^, was executed as the command “java -Xms 400 g -jar RNA-Bloom.jar -t 96 -outdir rnabloom_out –long fastq_list.txt.” The input data were the pre-processed reads (FASTQ files). For the TDP-43-related samples, the reads were reduced by half using SeqKit^67^ (“seqkit sample -p 0.5” command) because of a memory-usage issue. The assembled contigs were mapped to the reference genome using GMAP^68^ (v2023-07-20) with the options “-t 96 -f 2 -n 1 --no-chimeras --min-trimmed-coverage=0.5 --min-identity=0.95.” The resulting GFF file was converted into a GTF file using GffRead^41^. The isoform structures with introns of 100 kbp or more were excluded using the in-house script. Note that the options of GMAP related to intron length (*e.g*., --max-intronlength-middle) were not effective to limit those lengths. After filtering of isoform structures, the final GTF file was generated.

### Flair execution

Flair^38^ was executed through the commands of “align --nvrna,” “correct,” and “collapse --generate_map.” The inputs were the mapped nanopore reads (BAM files), reference genome, and reference annotation.

### FLAMES execution

The “bulk_long_pipeline.py” script of FLAMES^37^ (v0.1) was executed with a config JSON file of parameters. The parameters were derived from an example file in the GitHub repository of FLAMES except for “strand_specific.” The config JSON file is as follows:

{“pipeline_parameters”:{“do_genome_alignment”:true, “do_isoform_identification”:true, “do_read_realignment”:true, “do_transcript_quantification”:true}, “global_parameters”:{“generate_raw_isoform”:true, “has_UMI”:false},“isoform_parameters”:{“MAX_DIST”:10, “MAX_TS_DIST”:100, “MAX_SPLICE_MATCH_DIST”:10, “min_fl_exon_len”:40, “Max_site_per_splice”:3, “Min_sup_cnt”:10, “Min_cnt_pct”:0.01, “Min_sup_pct”:0.2, “strand_specific”:0, “remove_incomp_reads”:5}, “alignment_parameters”:{“use_junctions”:true, “no_flank”:true}, “realign_parameters”:{“use_annotation”:true}, “transcript_counting”:{“min_tr_coverage”:0.75, “min_read_coverage”:0.75}}.

### TALON execution

TALON^36^ (v6.0.1) was executed through the commands of “talon_initialize_database,” “talon_label_reads,” “talon,” “talon_create_GTF,” and “talon_filter_transcripts.” The specified options were “--ar 20” of “talon_label_reads” and “--maxFracA 0.5 --minCount 5 –minDatasets 1” of “talon_filter_transcripts.” These values were derived from the example commands in the GitHub repository of TALON. Input reads were converted to reverse complement sequences if they were aligned to the minus strands of the reference transcripts, because TALON can not handle reverse complement reads. The processed reads were aligned to the reference genome using Minimap2^63^ with the options of “--MD -a -x splice -ub -k14.” The resulting BAM file was input into the TALON commands.

### Scallop2 execution

Scallop2^39^ (v1.1.2) was executed with the default parameters. The inputs were the short reads mapped by STAR^54^, reference genome, and reference annotation.

### Cufflinks execution

The reference genome was indexed using the “bowtie2-build” command of Bowtie2^69^ (v2.2.5) with the default parameters. Short reads were mapped using TopHat2^70^ (v2.1.1) to the Bowtie2-indexed genome with the reference annotation and the default parameters. Cufflinks^40^ (v2.2.1) was executed with TopHat2-mapped reads (BAM files), the reference annotation, and the default parameters.

### SQANTI3 execution

To evaluate transcript structure predictions based on the number of short reads supporting splice junctions, the “sqanti3_qc.py” script of SQANTI3^45^ (v5.3.5) was executed with the options of “-d sqanti3_qc_out,” “-o out,” “--force_id,”_”ignore --skipORF,” and “--cpus 32.” The inputs were the reference genome, the reference annotation, and short reads mapped to the reference genome by STAR^54^ (BAM files). The numbers of splice junctions supported by at least one short read were counted using an in-house script based on the file of “sqanti3_qc_fastq_out/out_junctions.txt.”

### SUPPA execution

The “suppa.py” script of SUPPA^21^ (v2.3) was used to detect differential splicing events between TDP-43 knockdown and scramble samples of iPS-derived motor neurons. Input data were CPMs and TPMs (transcripts per million) of transcripts for long and short reads, respectively. The long-read CPMs and short-read TPMs were calculated using an in-house script with Minimap2^63^ and RSEM^25^ with STAR^54^, respectively. The execution of SUPPA consisted of three steps: (1) The “generateEvents” subcommand was executed with the option of “-e SE SS MX RI FL” and the reference annotation. (2) The “psiPerEvent” subcommand was executed with the output of step (1) and TPM or CPM data. This step was executed for each type of event (“SE”, “RI,” “MX,” “AL,” “AF,” “A5,” or “A3”). (3) The “diffSplice” subcommand was executed with the output of the step (2) and the option of “-gc.” The data of the knock-down and control samples were input separately for comparison. This step was also executed for each type of event (“SE”, “RI,” “MX,” “AL,” “AF,” “A5,” or “A3”). Note that the reference annotations (GTF file) were the result from IsoRefiner and GENCODE v42 for Supplementary Fig. 7B and 7C, respectively.

### Computational resources

We utilized the AWS cloud computing environment, using three types of instances: m5.8xlarge (32 vCPU, 128 GiB memory), c5a.24xlarge (96 vCPU, 192 GiB memory), and hpc6a.48xlarge (96 CPU, 384 GiB memory). We conducted parallel computing using ParallelCluster (v3.3.1) with up to 10 nodes.

### Statistics and reproducibility

Sample sizes were determined based on a balance between experimental feasibility and the need for sufficient data to support RNA-seq analysis and downstream computational tool development. All statistical analyses were performed using python (v3.9.21) and R (v4.3.2).

### Reporting summary

Further information on research design is available in the Nature Portfolio Reporting Summary linked to this article.

## Supplementary information

Transparent Peer Review file

Supplementary information

Description of additional supplementary data

Supplementary data 1

Supplementary data 2

Supplementary data 3

Supplementary data 4

Supplementary data 5

Supplementary data 6

Supplementary data 7

Supplementary data 8

Supplementary data 9

Supplementary data 10

Supplementary data 11

Reporting Summary

## Data Availability

All of our sequence data have been deposited in the DDBJ/ENA/GenBank Sequence Read Archive under BioProject ID PRJDB19918. The public RNA-seq datasets are available under accession number GSE121569^12^ (iPSC-derived motor neurons), PRJEB42763^15^ (iPSC-derived cortical neurons, SK-N-BE(2), and SH-SY5Y), and GSE126543^47^ (patients with ALS and FTD). The source data for this study are available in Supplementary Data 4–11.

## Code availability

Our developed method, IsoRefiner for long-read transcriptome analysis is available at https://github.com/rkajitani/IsoRefiner.
