# Supplementary material for: Long-read RNA sequencing unveils a novel cryptic exon in MNAT1 along with its full-length transcript structure in TDP-43 proteinopathy
Source: Commun Biol. 2025 Jul 16;8:1056. doi: 10.1038/s42003-025-08463-4 (PMC12267460; doi:10.1038/s42003-025-08463-4)
Supplement: Supplementary file 2 — Supplementary information [file 42003_2025_8463_MOESM2_ESM.pdf]

Supplementary Information

Title:

Long-read RNA sequencing unveils a novel cryptic exon in *MNAT1* along with its full-length transcript structure in TDP-43 proteinopathy

Authors:

Yoshihisa Tanaka, Naohiro Sunamura, Rei Kajitani, Marie Ikeguchi, Ryo Kunimoto*

(*Corresponding author: ryo.kunimoto@daiichisankyo.com)

Affiliation:

Research Innovation Planning Department, Research & Development Division, Daiichi Sankyo Co., Ltd., Tokyo, Japan

This file includes:

Supplementary Figures 1 to 12

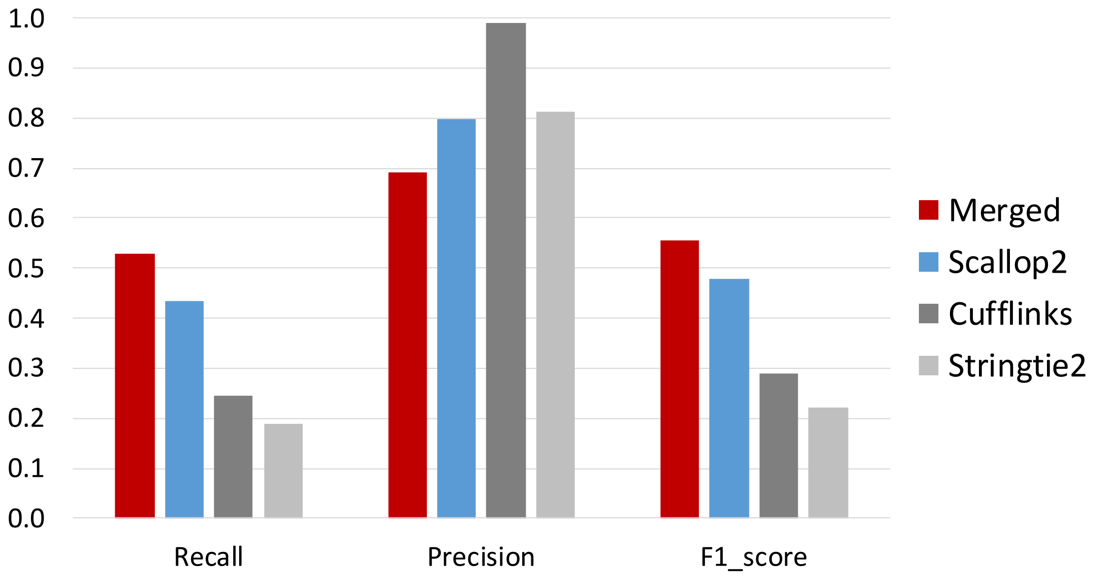

Supplementary Figure 1: Evaluation of short-read analysis tools for simulation data. Performance evaluation of several long-read analysis tools using simulation datasets. Metrics for recall, precision, and F1 score are defined in Method.

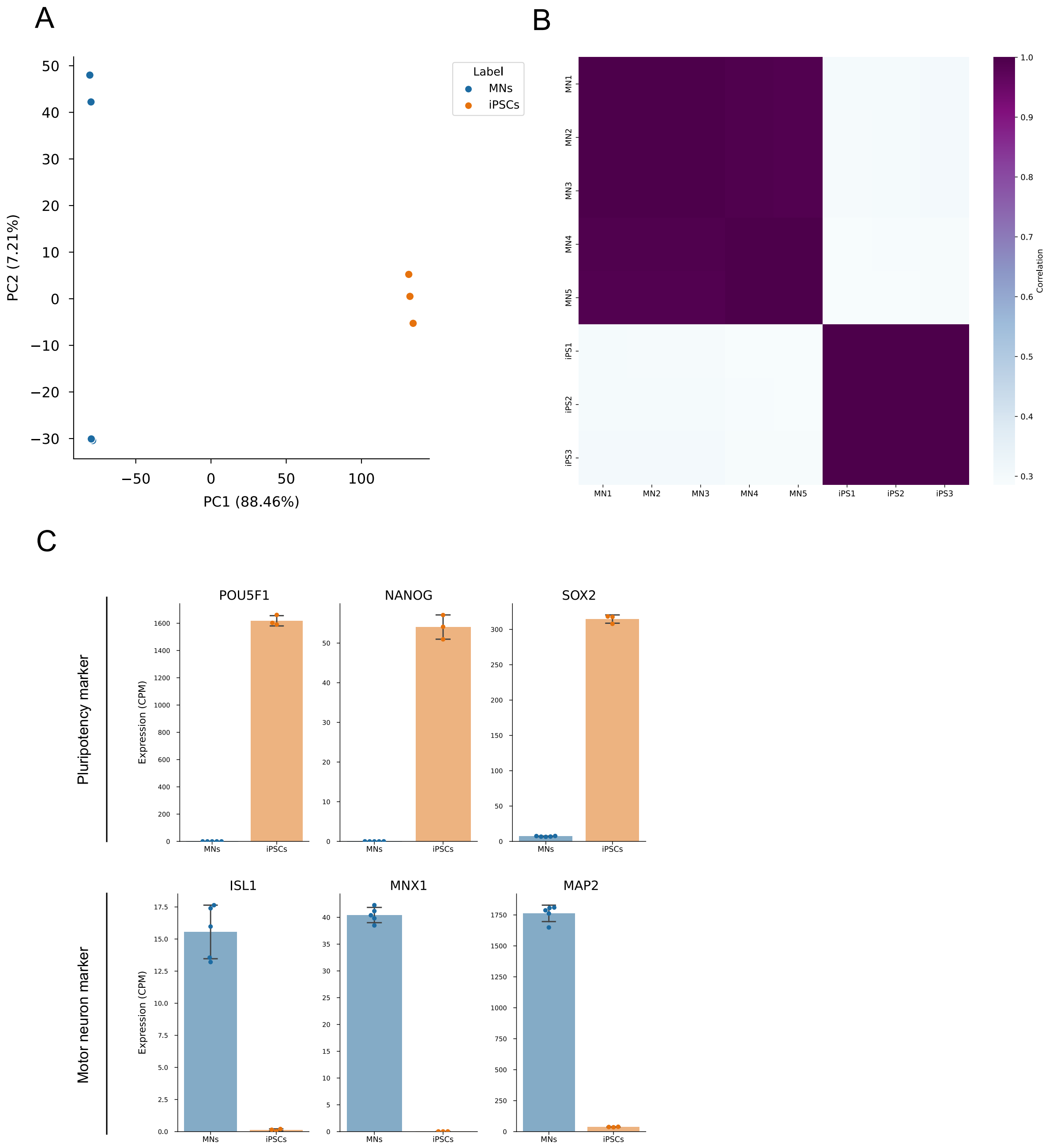

Supplementary Figure 2: Evaluation of motor neuron differentiation. (**A, B**) PCA and correlation analysis in iPSCs (*n*=3, orange) and motor neurons (MNs; *n*=5, blue) using short-read RNA-seq. (**C**) Quantification of mRNA expression of marker genes for both pluripotency and motor neurons in iPSCs (*n*=3, orange) and MNs (*n*=5, blue). Column and error bars represent the mean and standard deviation, respectively. CPM, count per million.

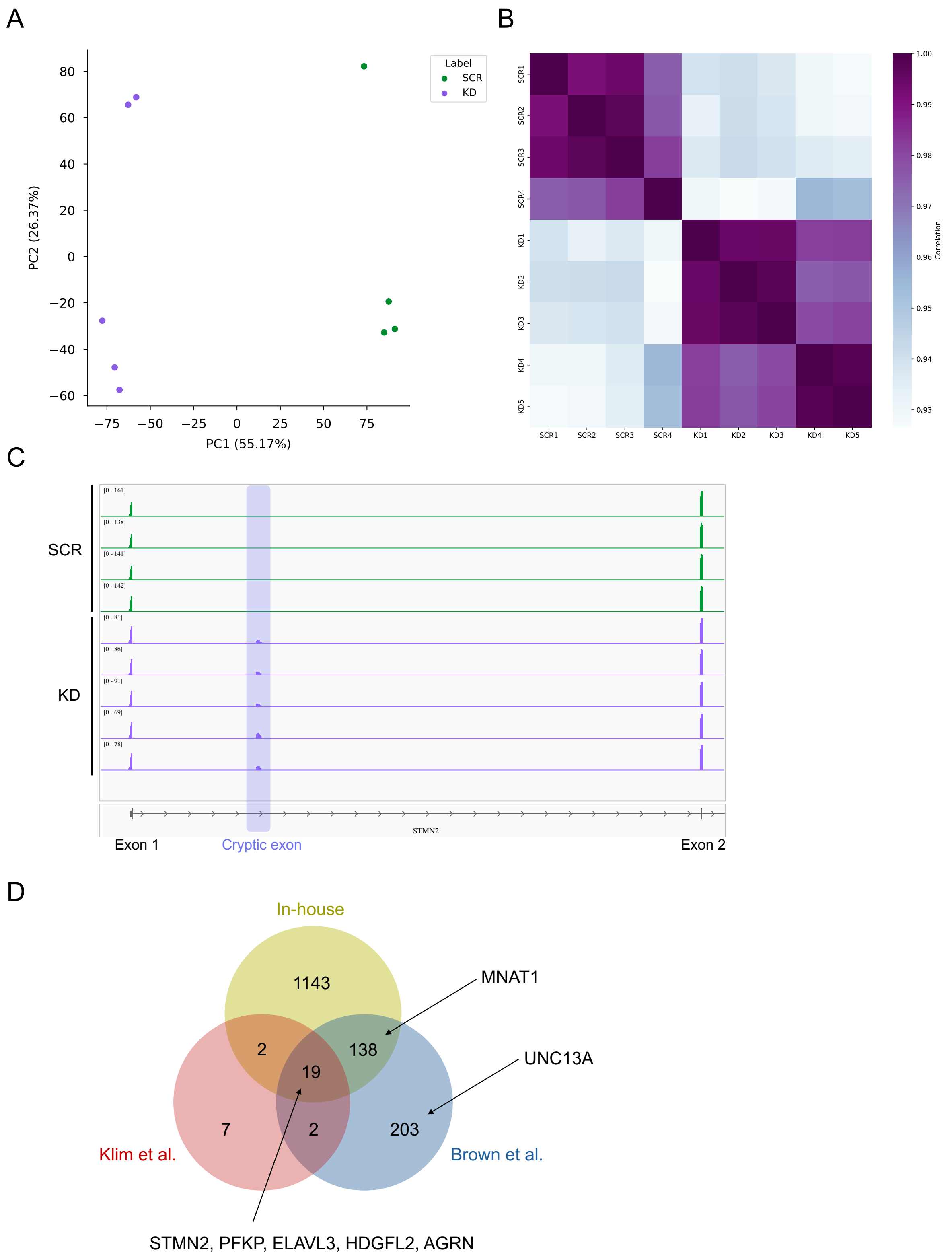

Supplementary Figure 3: Evaluation of TDP-43 knockdown in motor neurons. (**A, B**) PCA and correlation analysis in motor neurons under scramble (SCR; *n*=4, green) and TDP-43 knockdown (KD; *n*=5, purple) conditions using short-read RNA-seq. (**C**) Visualization of the *STMN2* cryptic exon in scramble and knockdown motor neurons. (**D**) Venn diagram

comparing genes exhibiting significantly differential splicing events (LeafCutter adjusted *p*-value < 0.05, unannotated) between control and TDP-43 knockdown conditions in in-house and external datasets (Klim et al.: iPSC-derived motor neurons; Brown et al.: iPSC-derived cortical-like neurons).

A

Raw read

Sample	# of reads	Total (bp)	Mean length (bp)	Median length (bp)	N50 length (bp)	Maximum length (bp)
SCR1	13,403,277	11,775,088,206	879	795	959	166,003
SCR2	19,043,572	17,140,466,649	900	801	997	198,475
SCR3	15,795,341	14,693,734,706	930	821	1,035	224,161
SCR4	14,540,026	14,171,623,345	975	847	1,092	291,888
KD1	8,001,914	7,137,229,903	892	796	986	186,964
KD2	22,895,453	19,320,931,986	844	770	980	253,555
KD3	12,156,218	10,652,038,229	876	789	971	465,981
KD4	10,604,963	10,399,118,440	981	845	1,079	156,273
KD5	22,181,778	21,280,212,140	959	829	1,053	330,079

Trimmed read

Sample	# of reads	Total (bp)	Mean length (bp)	Median length (bp)	N50 length (bp)	Maximum length (bp)
SCR1	12,998,296	9,339,761,378	719	629	831	165,995
SCR2	18,463,845	13,850,502,783	750	645	886	198,475
SCR3	15,273,367	11,852,622,713	776	663	921	224,161
SCR4	14,148,630	11,640,627,452	823	691	977	291,881
KD1	7,731,631	5,692,715,203	736	634	874	186,955
KD2	20,298,541	14,905,066,335	734	630	875	253,548
KD3	11,687,933	8,472,540,528	725	630	857	465,919
KD4	10,377,664	8,537,718,749	823	685	960	156,265
KD5	21,698,237	17,269,994,346	796	664	935	330,079

B

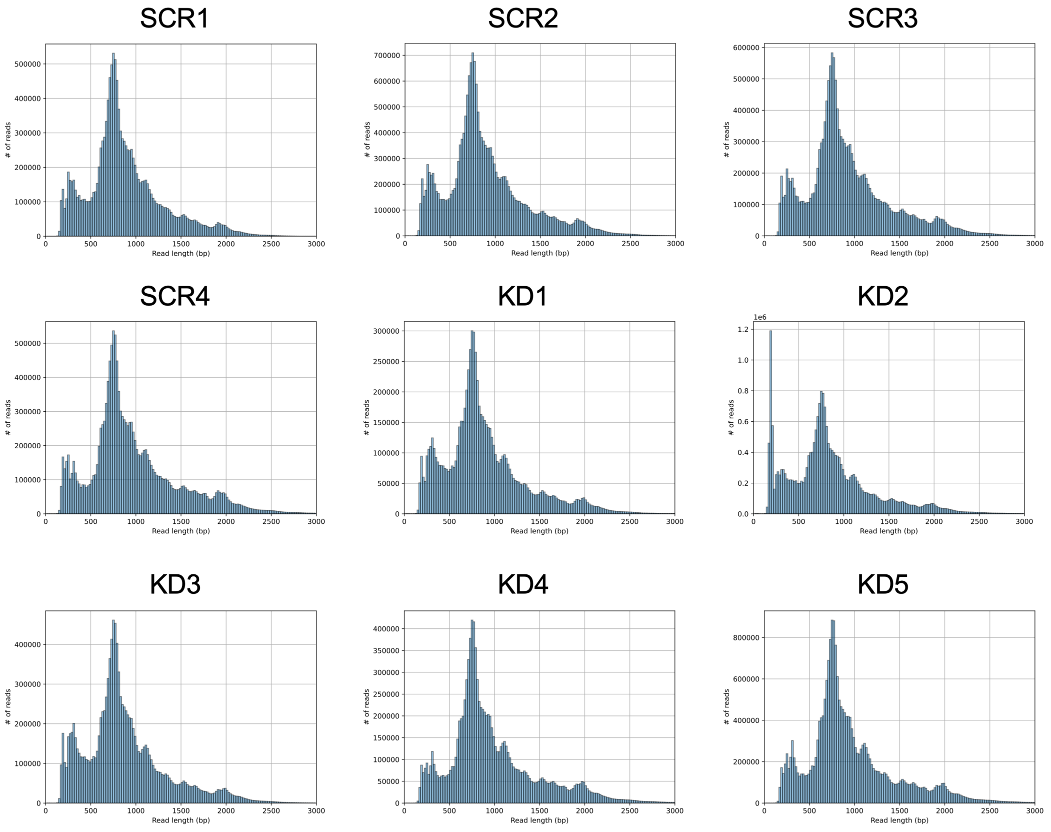

Supplementary Figure 4: Long-read RNA-seq metrics. (**A**) Summary of read statistics from long-read RNA-seq, including raw and trimmed reads. (**B**) Distribution of raw read lengths.

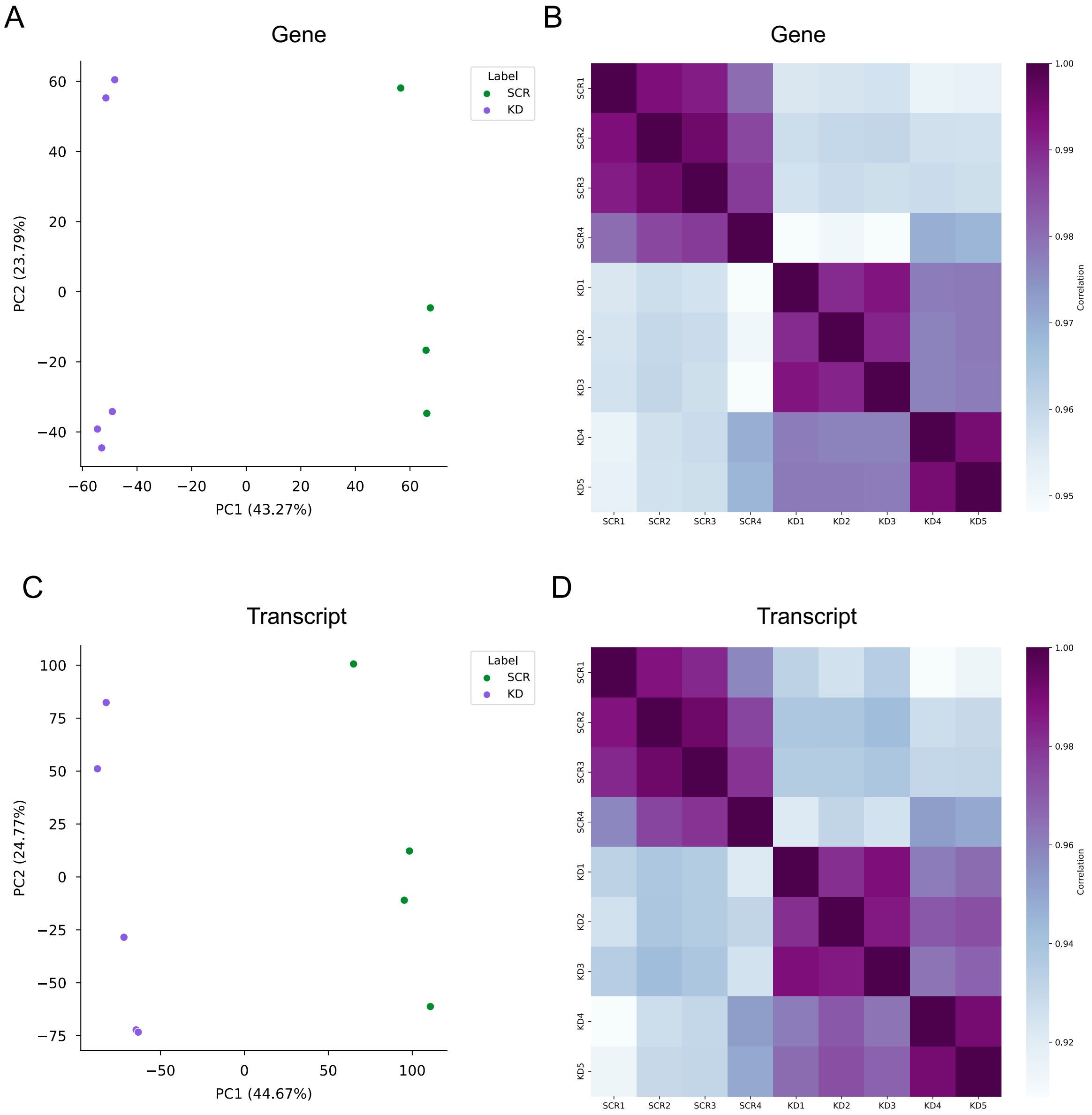

Supplementary Figure 5: Evaluation of long-read RNA sequencing. PCA and correlation analysis at the gene (**A, B**) and transcript (**C, D**) levels in motor neurons under SCR (*n*=4, green) and KD (*n*=5, purple) conditions, based on long-read RNA-seq.

A

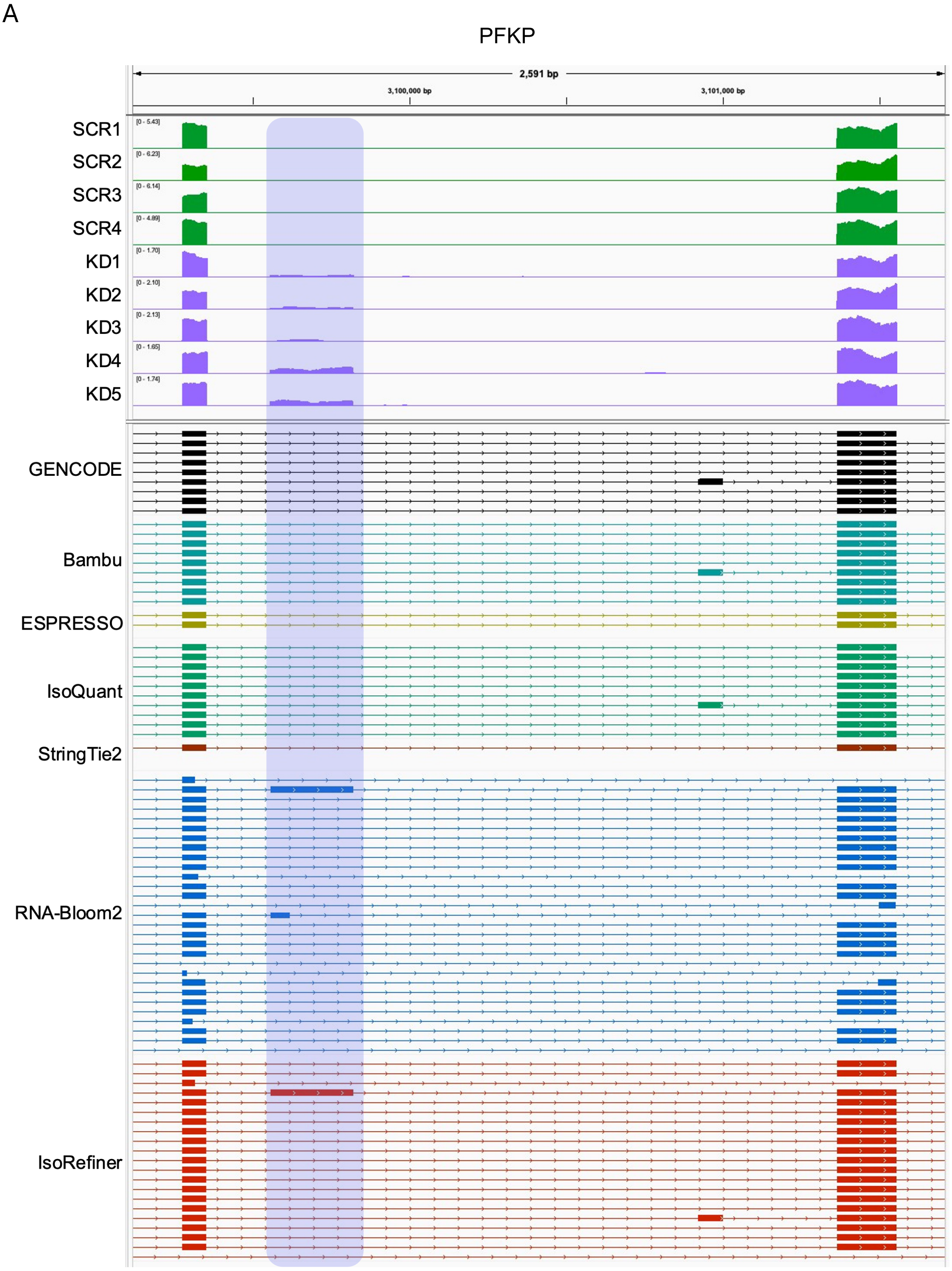

PFKP
2,591 bp
3,100,000 bp
3,101,000 bp
SCR1
SCR2
SCR3
SCR4
KD1
KD2
KD3
KD4
KD5
GENCODE
Bambu
ESPRESSO
IsoQuant
StringTie2
RNA-Bloom2
IsoRefiner

B-1

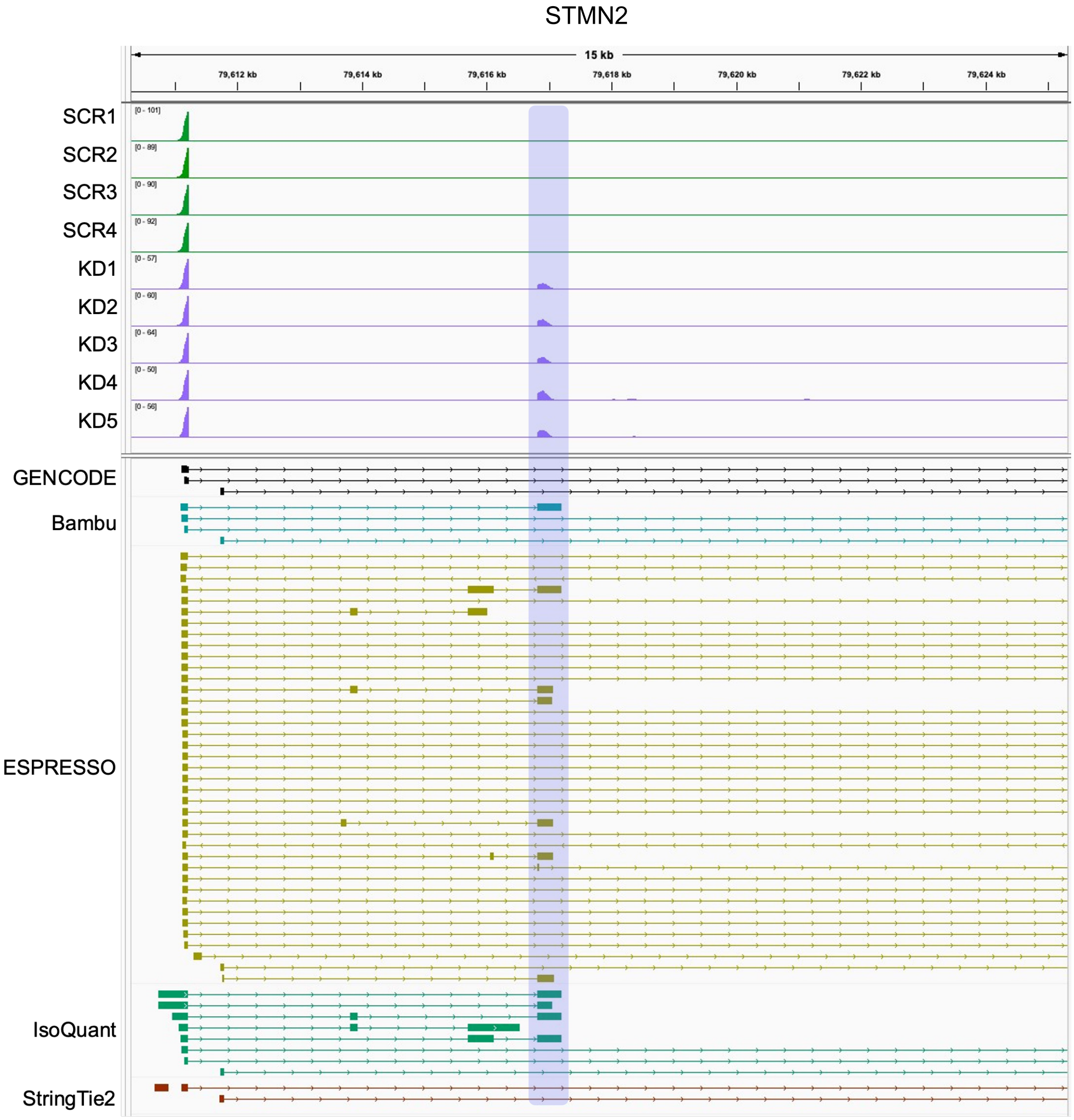
STMN2
15 kb
79,612 kb
79,614 kb
79,616 kb
79,618 kb
79,620 kb
79,622 kb
79,624 kb
SCR1
SCR2
SCR3
SCR4
KD1
KD2
KD3
KD4
KD5
GENCODE
Bambu
ESPRESSO
IsoQuant
StringTie2

B-2

STMN2

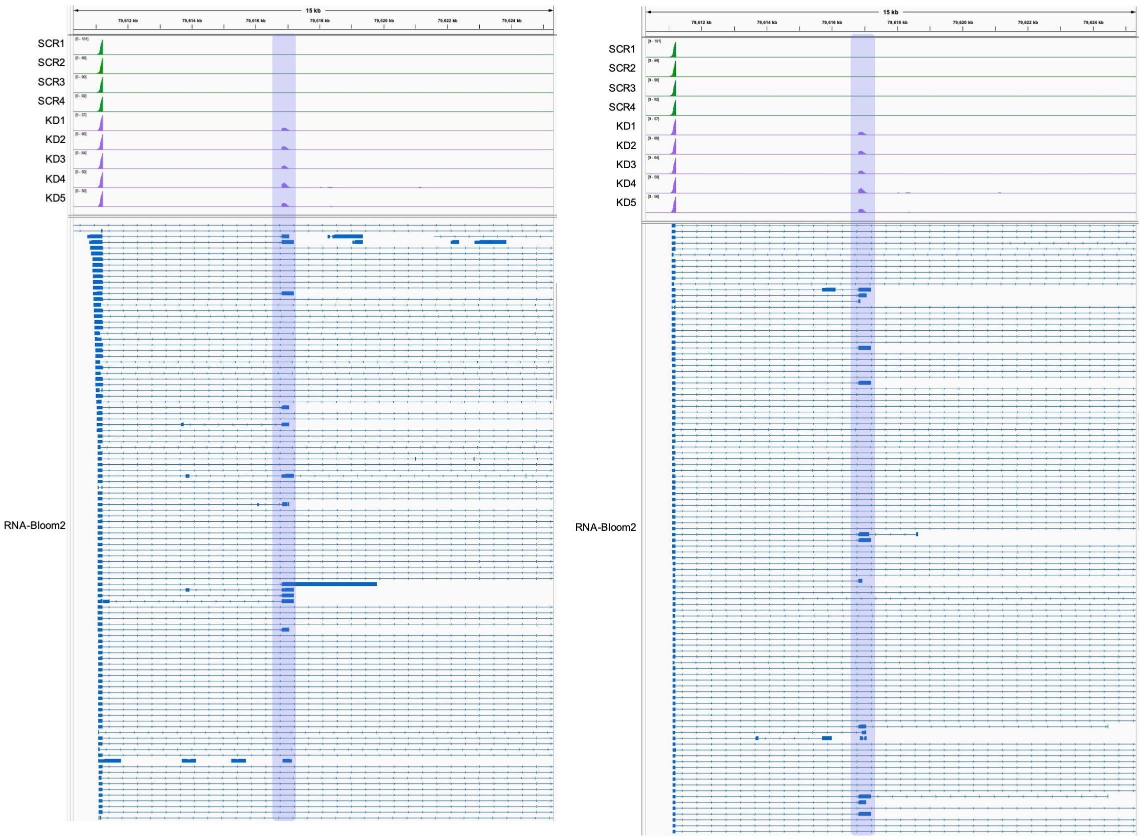

B-3

STMN2

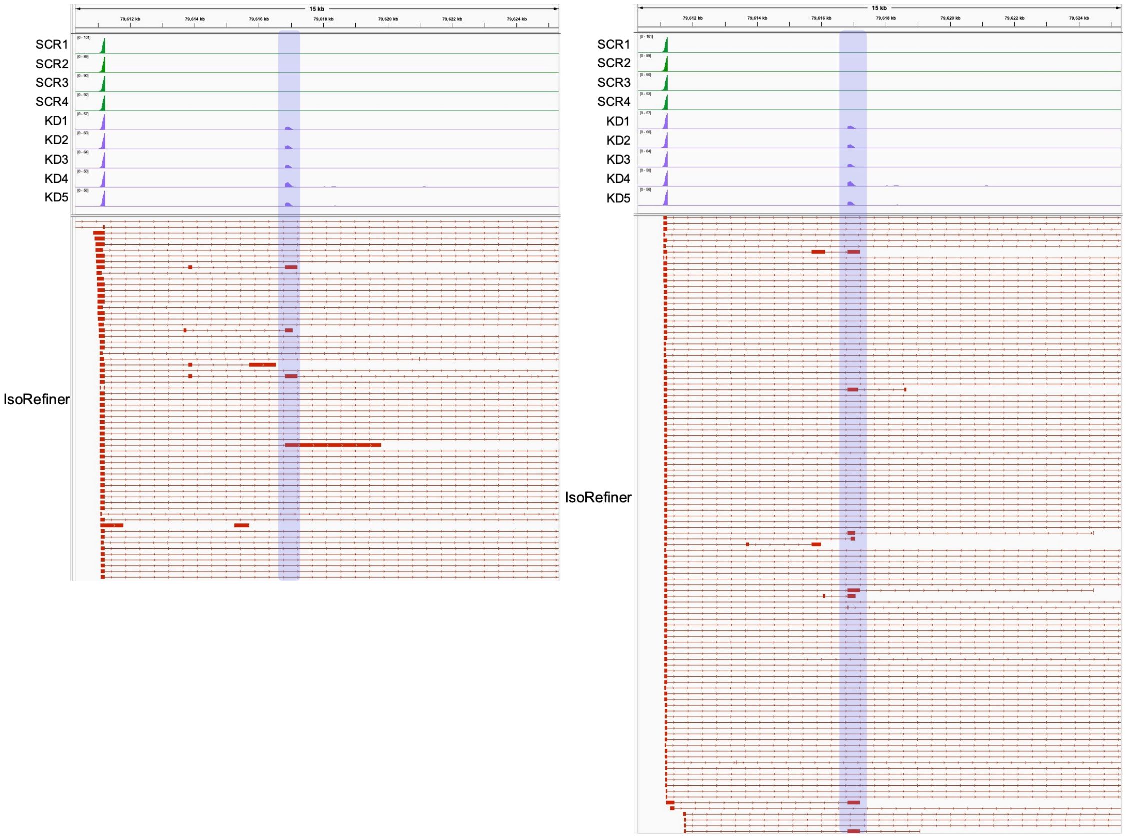

SCR1
SCR2
SCR3
SCR4
KD1
KD2
KD3
KD4
KD5
IsoRefiner
SCR1
SCR2
SCR3
SCR4
KD1
KD2
KD3
KD4
KD5
IsoRefiner

C

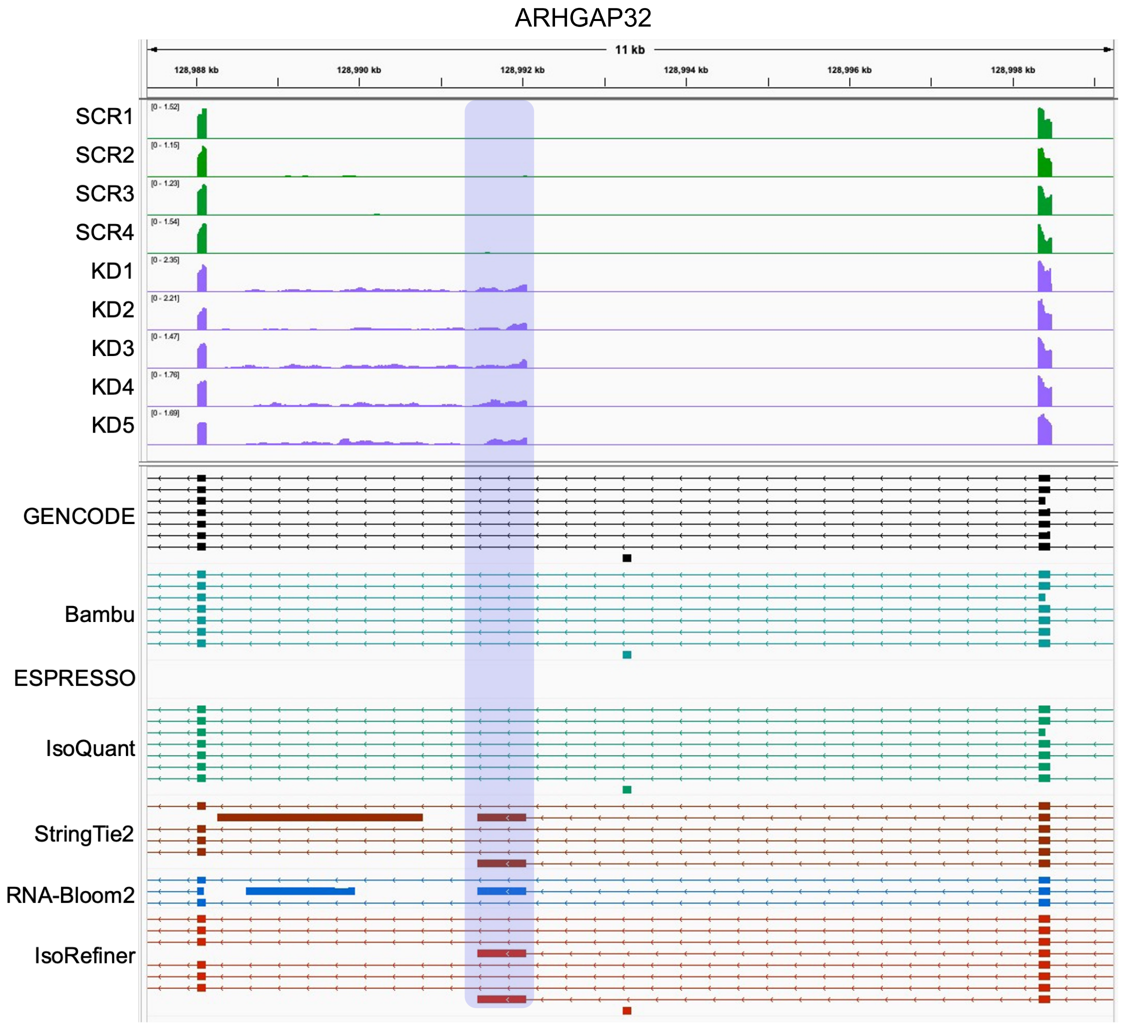
ARHGAP32
11 kb
128,988 kb
128,990 kb
128,992 kb
128,994 kb
128,996 kb
128,998 kb
SCR1
SCR2
SCR3
SCR4
KD1
KD2
KD3
KD4
KD5
GENCODE
Bambu
ESPRESSO
IsoQuant
StringTie2
RNA-Bloom2
IsoRefiner

D

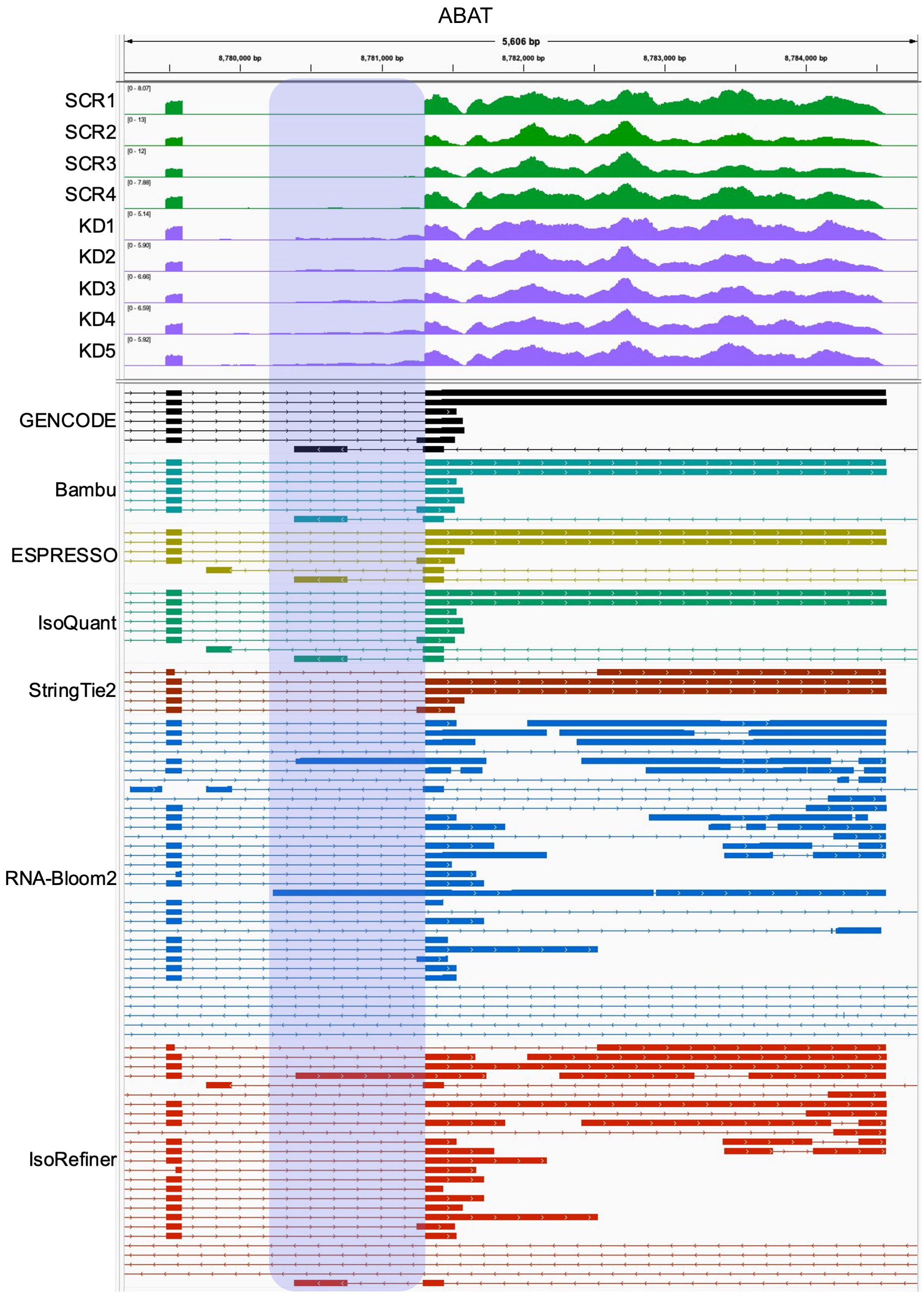
ABAT
5,606 bp
8,780,000 bp
8,781,000 bp
8,782,000 bp
8,783,000 bp
8,784,000 bp
SCR1
SCR2
SCR3
SCR4
KD1
KD2
KD3
KD4
KD5
GENCODE
Bambu
ESPRESSO
IsoQuant
StringTie2
RNA-Bloom2
IsoRefiner

E

ZC3H8

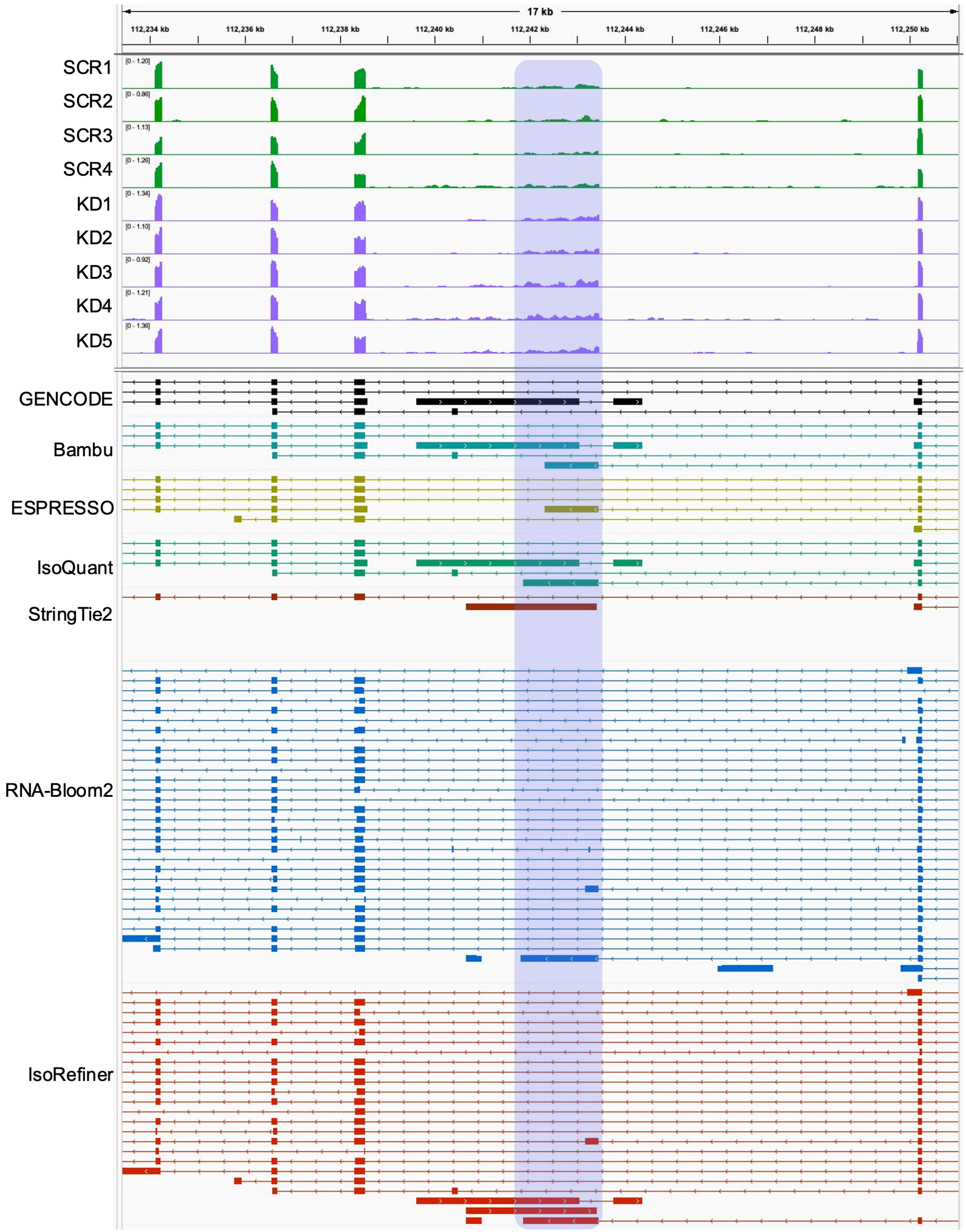
17 kb
SCR1
SCR2
SCR3
SCR4
KD1
KD2
KD3
KD4
KD5
GENCODE
Bambu
ESPRESSO
IsoQuant
StringTie2
RNA-Bloom2
IsoRefiner

F-1

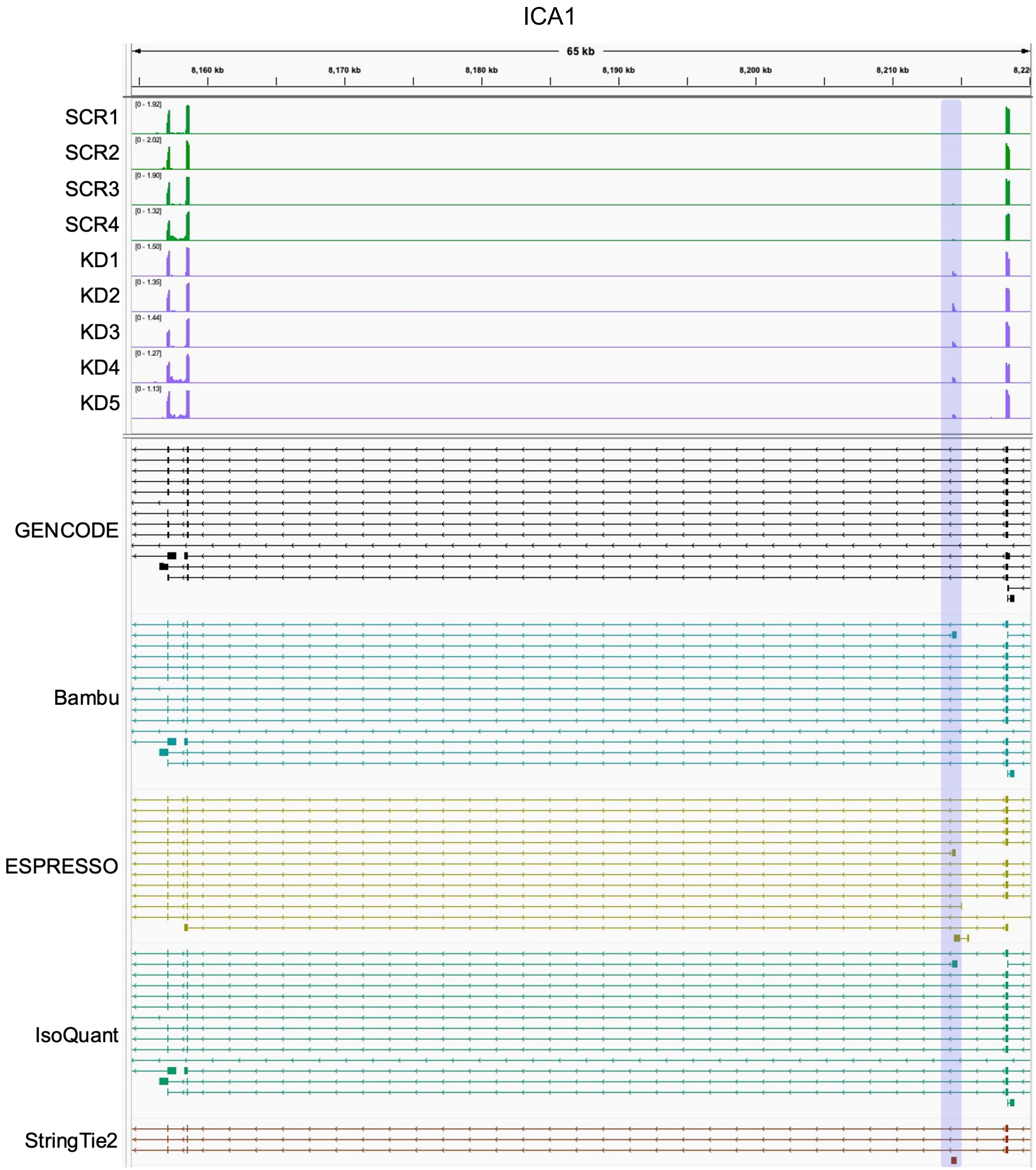
ICA1
65 kb
8,160 kb
8,170 kb
8,180 kb
8,190 kb
8,200 kb
8,210 kb
SCR1
SCR2
SCR3
SCR4
KD1
KD2
KD3
KD4
KD5
GENCODE
Bambu
ESPRESSO
IsoQuant
StringTie2

F-2

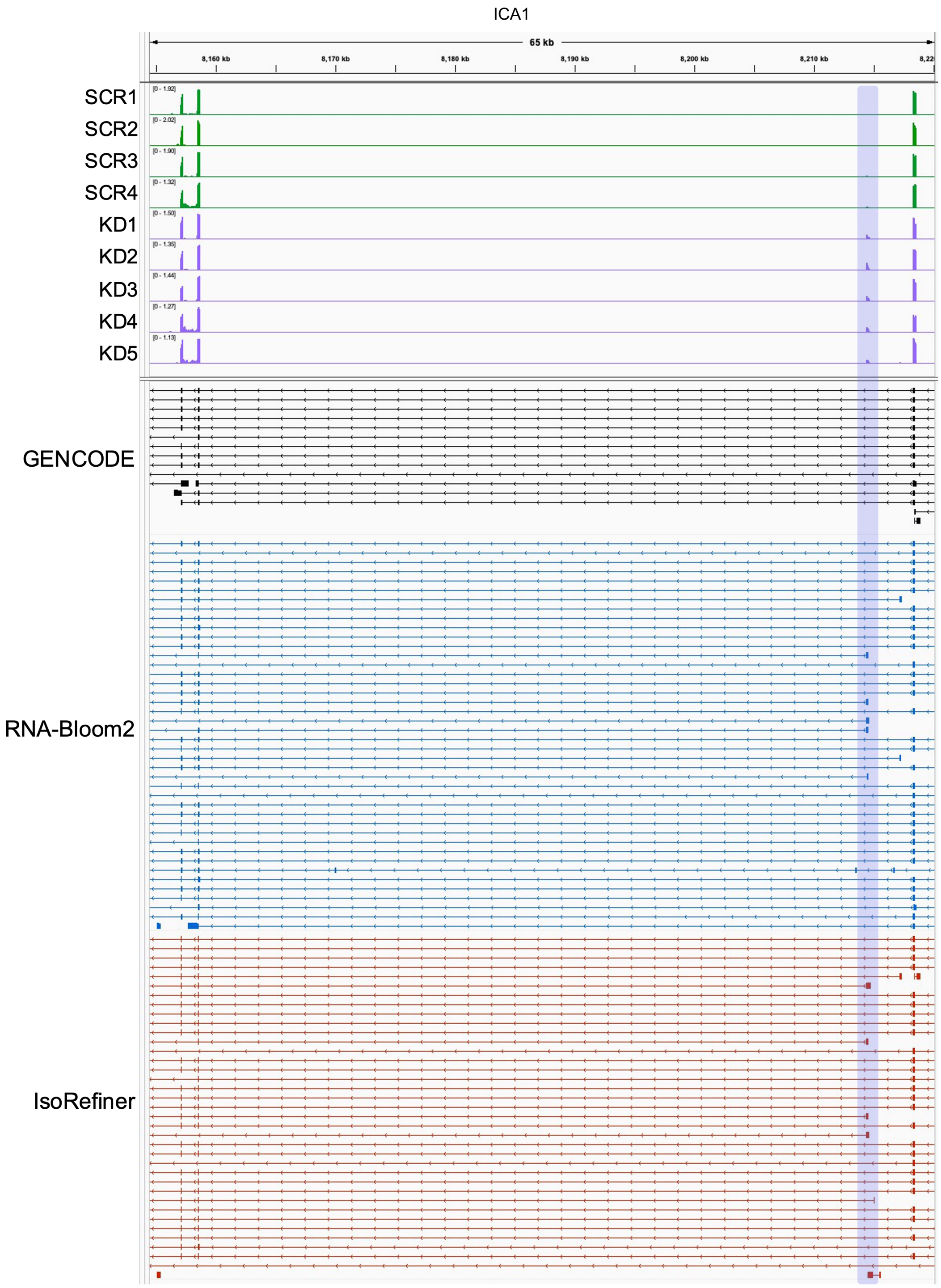

Supplementary Figure 6: Construction of transcript structures for the detected cryptic exons using short- and long-read RNA-seq. Read coverage tracks for SCR (*n*=4, green) and KD (*n*=5, purple) motor neurons were generated using short reads. The Y-axis represents CPM. The lower panel shows the transcript structures constructed by each long-read transcriptome analysis tool. The light blue region indicates the position of the cryptic exon. (**A**) *PFKP*, (**B**) *STMN2*, (**C**) *ARHGAP32*, (**D**) *ABAT*, (**E**) *ZC3H8*, and (**F**) *ICA1*.

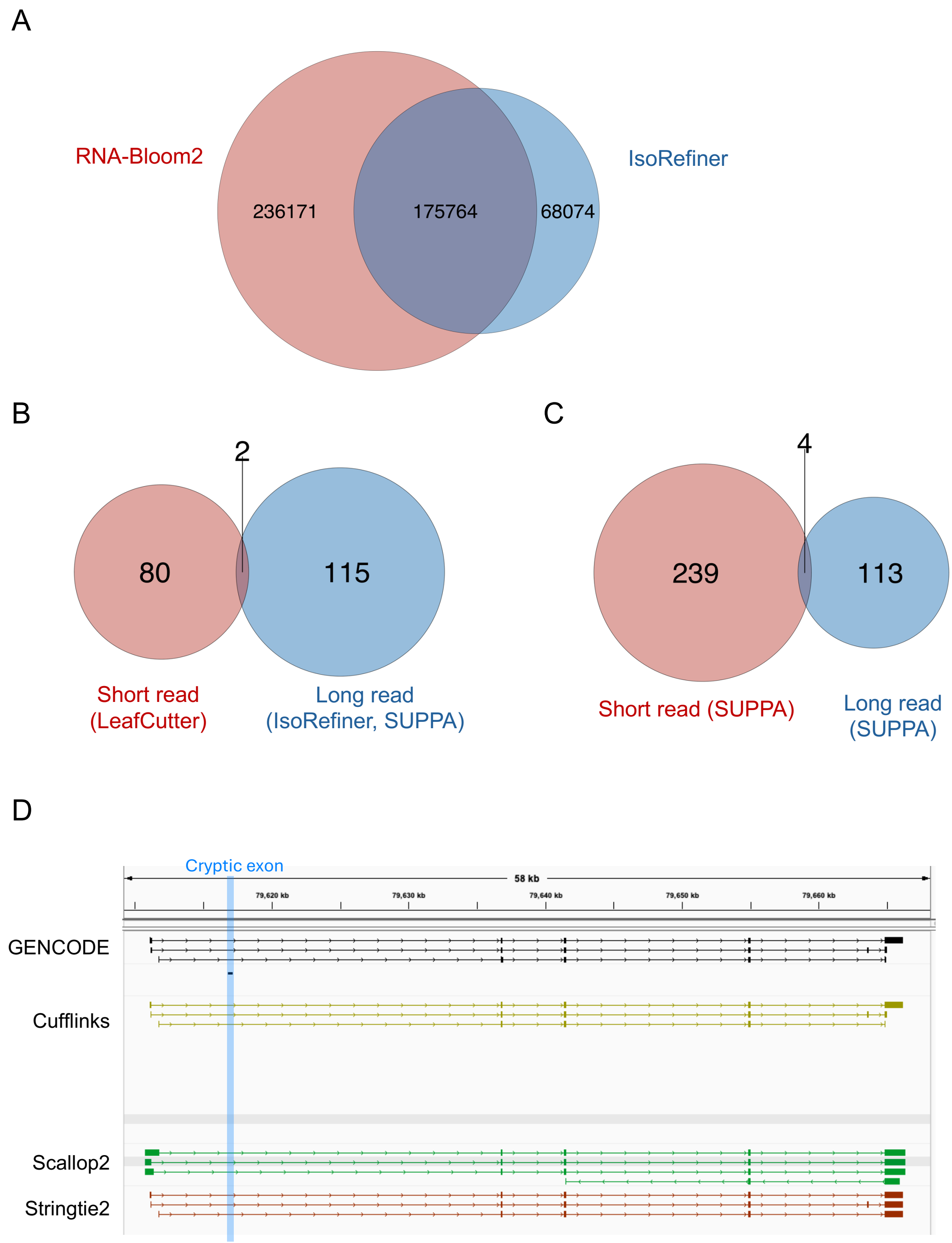

Supplementary Figure 7: Comparative evaluation of short- and long-read approaches for transcript structure reconstruction. (**A**) Venn diagram comparing transcript structure sets obtained from IsoRefiner and RNA-Bloom2, using long reads from motor neurons. The numbers inside the diagram indicate the intron-chain counts for novel transcript structures. (**B, C**) Venn diagram of genes exhibiting significantly differential splicing events in long- versus short-read data. Significance was defined by an adjusted p-value < 0.01 and $|\Delta PSI| \geq 0.1$ for comparisons between SCR and KD conditions. (**B**) Comparison between short-read result (LeafCutter) and long-read result (IsoRefiner and SUPPA). (**C**) Comparison between short- and long-read results (SUPPA), excluding novel transcripts. (**D**) *STMN2* transcript structures constructed from short reads using the respective tool.

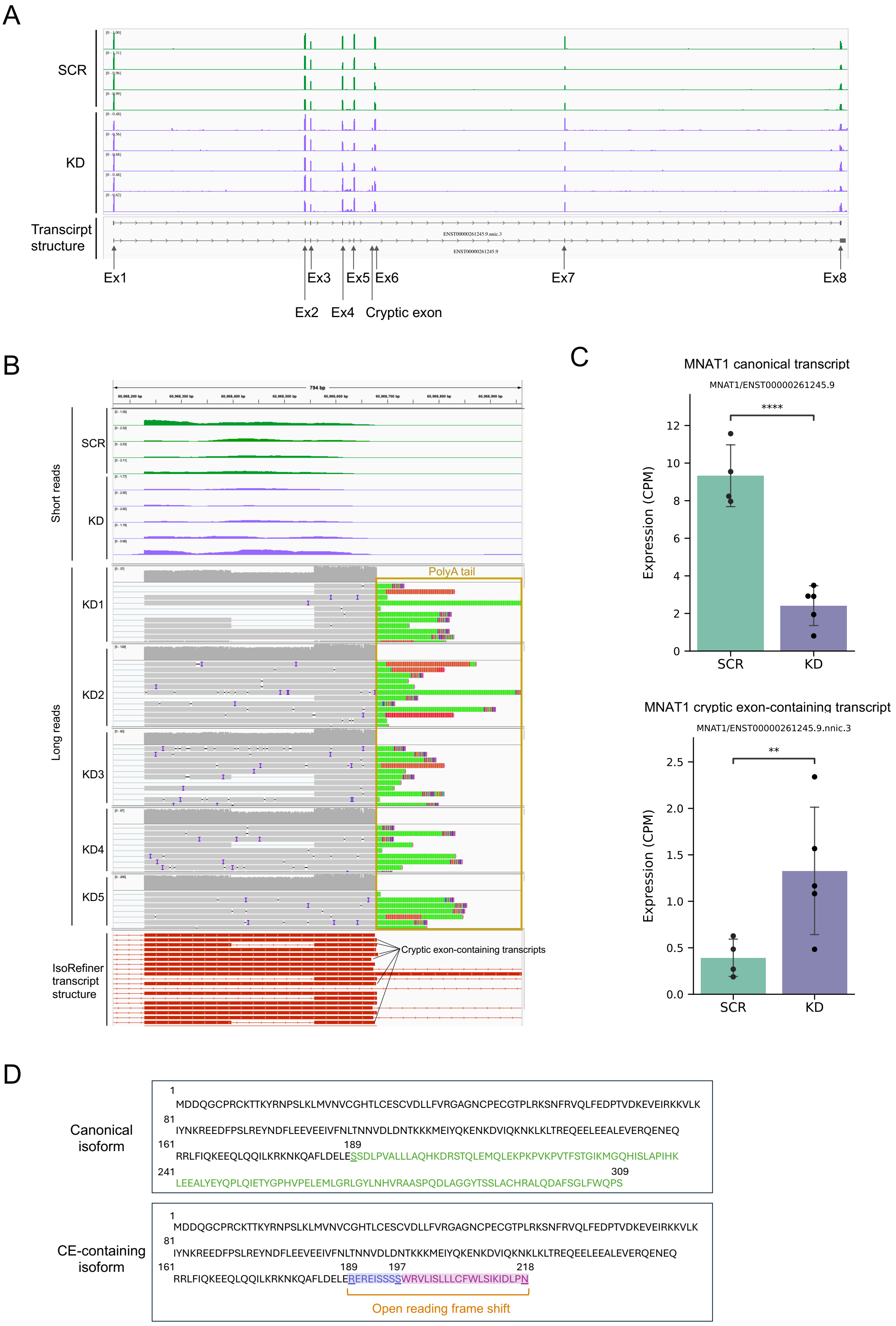

Supplementary Figure 8: Determination of the full-length cryptic exon-containing *MNAT1* transcript. (**A**) Full-length structures of both the canonical and cryptic exon-

containing *MNAT1* transcripts determined by long-read RNA-seq (bottom panel). Read coverage tracks for SCR (*n*=4, green) and KD (*n*=5, purple) motor neurons were generated using short reads. Ex, Exon. (**B**) Read visualization of polyA tails by long reads. (**C**) Comparison of the full-length mRNA expression of the canonical and cryptic exon-containing *MNAT1* transcripts between SCR (*n*=4, green) and KD (n=5, purple) motor neurons. Column and error bars represent the mean and standard deviation, respectively. **, adjusted *p*-value < 0.01: ****, adjusted *p*-value < 0.0001 (DESeq2). (**D**) ORF prediction of the *MNAT1* full-length transcript isoforms. The cryptic exon-induced frameshift on translation is highlighted in blue (cryptic exon, residues 189 to 197) and magenta (canonical exon 6, residues 198 to 218). Letters represent amino acids, and the number corresponds to the amino acid residue. CE, cryptic exon.

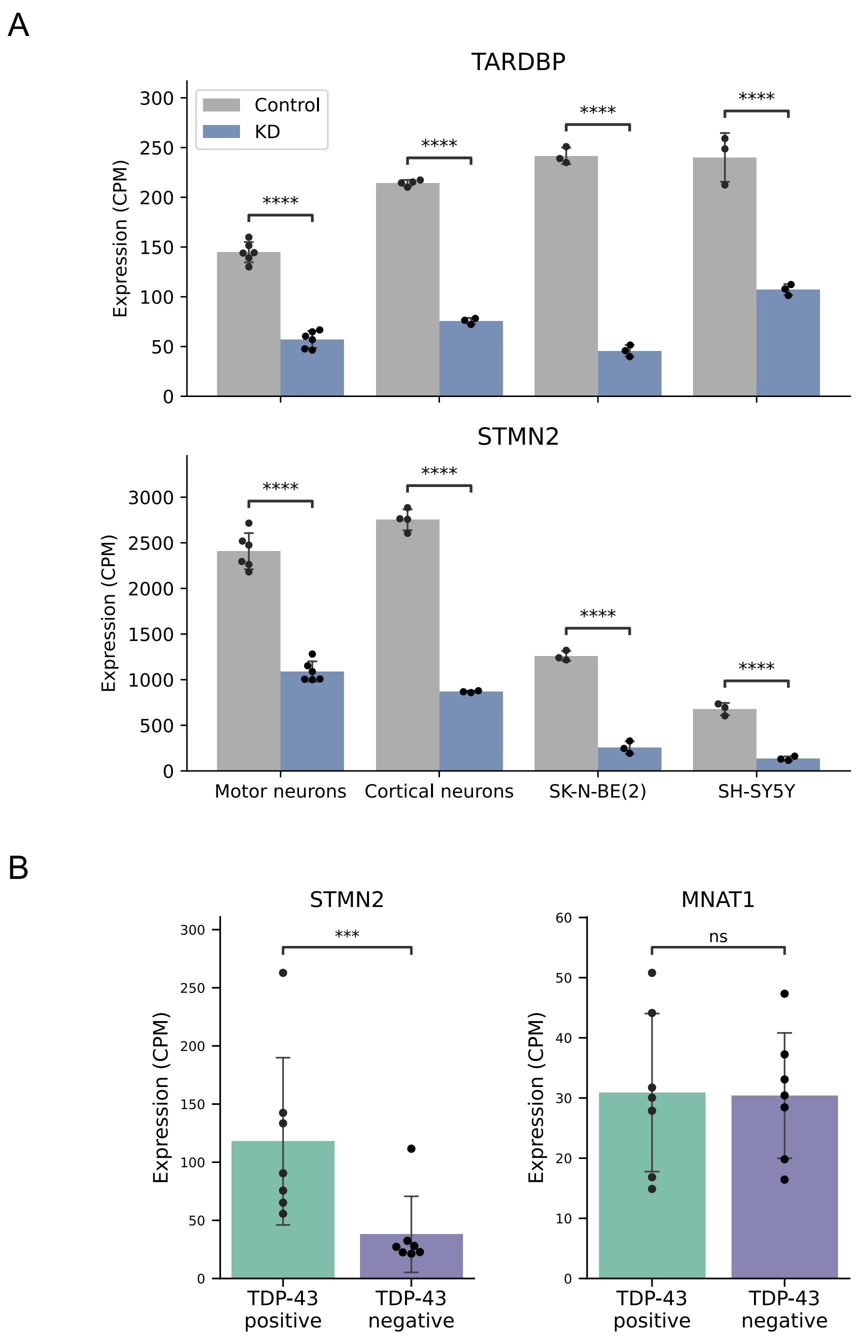

Supplementary Figure 9: Analysis of mRNA levels in TDP-43 dysfunctional neuronal models. (**A**) Quantification of *TARDBP* and *STMN2* mRNA expression under control and TDP-43 knockdown (KD) conditions across four cell types, using previously published datasets: iPSC-derived motor neurons (control and KD each, n=6), iPSC-derived cortical neurons (control, n=4; KD, n=3), and SK-N-BE(2) and SH-SY5Y (control and KD each, n=3). (**B**) Quantification of *MNAT1* mRNA expression in TDP-43-positive (n=7) and TDP-43-negative (n=7) nuclei samples. ***, adjusted p-value < 0.001: ****, adjusted p-value < 0.0001: ns, not significant (DESeq2). Column and error bars represent the mean and standard deviation, respectively. CPM, count per million.

A

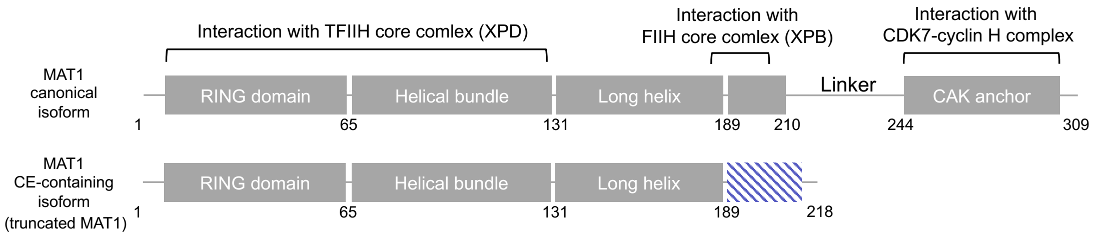

B

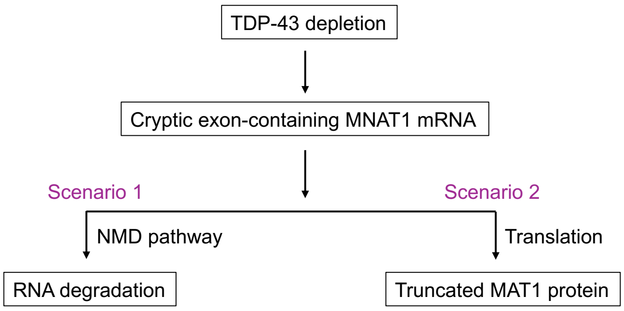

Supplementary Figure 10: Proposed consequences of the *MNAT1* cryptic exon on transcription and translation. (**A**) Illustration of the MAT1 protein isoforms, highlighting functional domains. The number corresponds to the amino acid residue with the N-terminus on the left and the C-terminus on the right. (**B**) Two proposed scenarios illustrating how the *MNAT1* cryptic exon may affect transcription and translation.

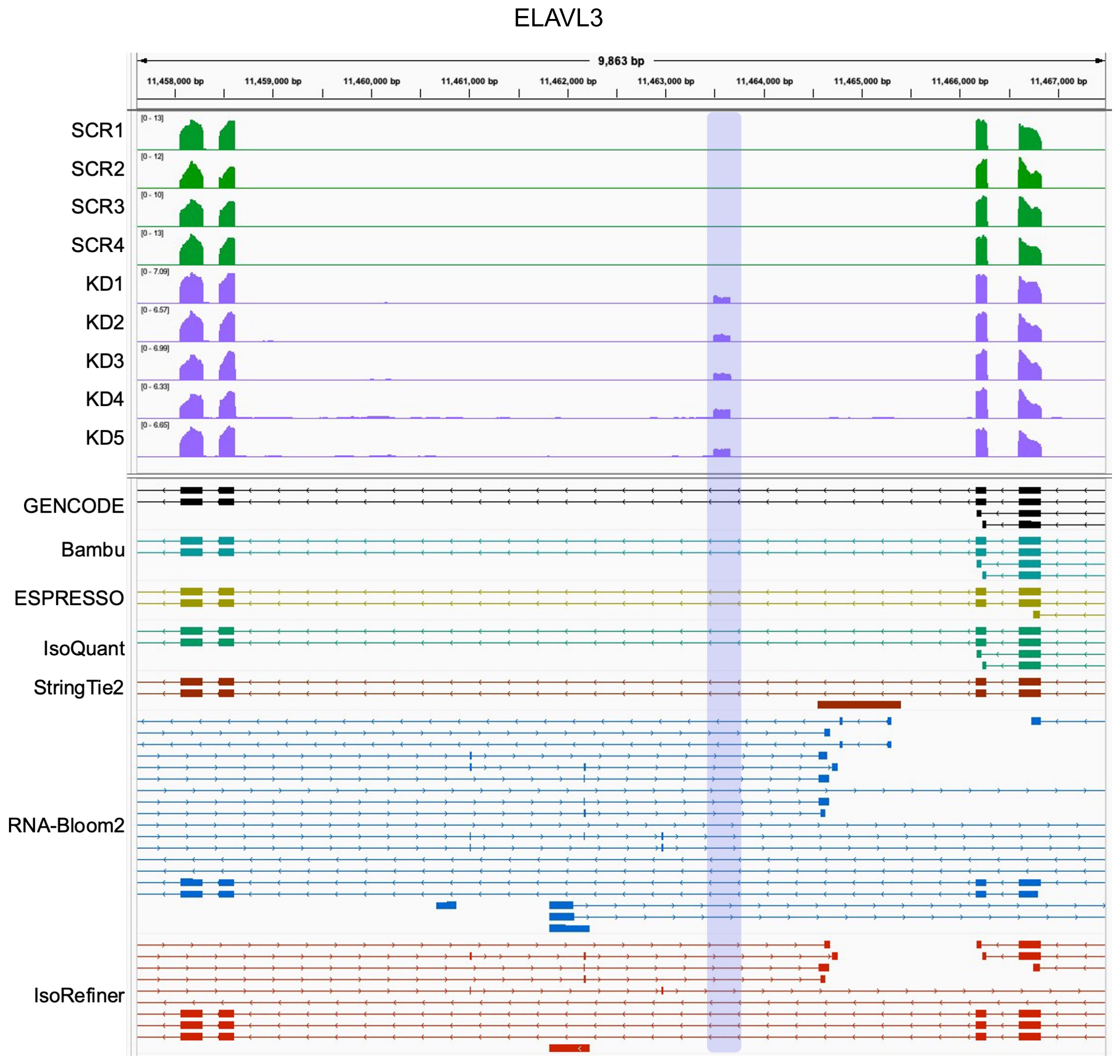

Supplementary Figure 11: Visualization of the *ELAVL3* cryptic exon predicted by short reads. Read coverage tracks for SCR (*n*=4, green) and KD (*n*=5, purple) motor neurons generated by short reads indicate a cryptic exon in the *ELAVL3* gene, while the long-read RNA-seq fails to construct transcript structures (lower panel) at this cryptic exon, regardless of the tools used. The Y-axis represents CPM.

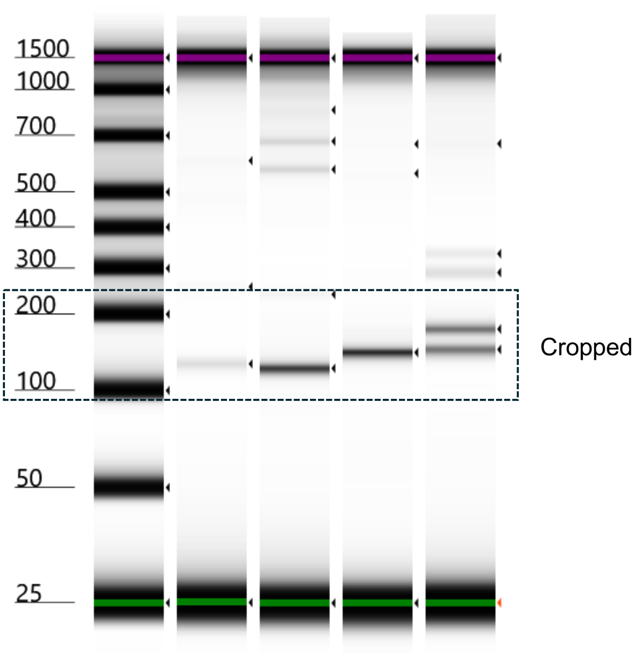

Supplementary Figure 12: Uncropped raw data for this study. Related to Fig. 5C.
