# Supplementary material for: Long-read RNA sequencing unveils a novel cryptic exon in MNAT1 along with its full-length transcript structure in TDP-43 proteinopathy
Source: Commun Biol. 2025 Jul 16;8:1056. doi: 10.1038/s42003-025-08463-4 (PMC12267460; doi:10.1038/s42003-025-08463-4)
Supplement: Supplementary file 3 — Description of additional supplementary data [file 42003_2025_8463_MOESM3_ESM.pdf]

Description of Supplementary Data

Supplementary Data 1: Comprehensive list of the identified splicing junctions.

Supplementary Data 2: Sample information of the public datasets.

Supplementary Data 3: GTF file generated by IsoRefiner using our long-read RNA-seq.

Supplementary Data 4: Numerical source data for graphs related to simulation evaluations (Fig. 1 and Supplementary Fig. 1).

Supplementary Data 5: Numerical source data for Venn diagrams related to long- and short-read comparisons (Supplementary Fig. 7).

Supplementary Data 6: Short-read RNA-seq source data from in-house experiments.

Supplementary Data 7: Long-read RNA-seq source data from in-house experiments.

Supplementary Data 8: Short-read RNA-seq source data from the public datasets.

Supplementary Data 9: Source data for Venn diagrams illustrating spliced gene comparisons between in-house and public datasets (Supplementary Fig. 3D).

Supplementary Data 10: Splicing analysis results derived from in-house short-read RNA-seq (Fig. 3).

Supplementary Data 11: Source data for PSI calculations, including both in-house and public datasets.
