# Supplementary material for: Long-read RNA sequencing unveils a novel cryptic exon in MNAT1 along with its full-length transcript structure in TDP-43 proteinopathy
Source: Commun Biol. 2025 Jul 16;8:1056. doi: 10.1038/s42003-025-08463-4 (PMC12267460; doi:10.1038/s42003-025-08463-4)
Supplement: Supplementary file 15 — Reporting Summary [file 42003_2025_8463_MOESM15_ESM.pdf]

nature portfolio

Corresponding author(s): Ryo Kunimoto, COMMSBIO-25-0477

Last updated by author(s): Jun 27, 2025

Reporting Summary

Nature Portfolio wishes to improve the reproducibility of the work that we publish. This form provides structure for consistency and transparency in reporting. For further information on Nature Portfolio policies, see our Editorial Policies and the Editorial Policy Checklist.

Statistics

For all statistical analyses, confirm that the following items are present in the figure legend, table legend, main text, or Methods section.

n/a	Confirmed	
☐	☒	The exact sample size (*n*) for each experimental group/condition, given as a discrete number and unit of measurement
☐	☒	A statement on whether measurements were taken from distinct samples or whether the same sample was measured repeatedly
☐	☒	The statistical test(s) used AND whether they are one- or two-sided<br>*Only common tests should be described solely by name; describe more complex techniques in the Methods section.*
☒	☐	A description of all covariates tested
☒	☐	A description of any assumptions or corrections, such as tests of normality and adjustment for multiple comparisons
☐	☒	A full description of the statistical parameters including central tendency (e.g. means) or other basic estimates (e.g. regression coefficient) AND variation (e.g. standard deviation) or associated estimates of uncertainty (e.g. confidence intervals)
☐	☒	For null hypothesis testing, the test statistic (e.g. *F*, *t*, *r*) with confidence intervals, effect sizes, degrees of freedom and *P* value noted<br>*Give P values as exact values whenever suitable.*
☒	☐	For Bayesian analysis, information on the choice of priors and Markov chain Monte Carlo settings
☒	☐	For hierarchical and complex designs, identification of the appropriate level for tests and full reporting of outcomes
☐	☒	Estimates of effect sizes (e.g. Cohen's *d*, Pearson's *r*), indicating how they were calculated

Our web collection on statistics for biologists contains articles on many of the points above.

Software and code

Policy information about availability of computer code

Data collection	sra-tools (v2.10.0)

Data analysis	Trimming, mappping, counting, and differentially expressed gene analysis of short reads were performed by fastp (v0.20.1), STAR (v2.7.10b), RSEM (v1.3.1), DEseq2 (v1.42.0), respectively.<br>Mapped short reads (BAM files) were processed and visualized by Samtools (v1.6), deepTools (v3.5.5), and Integrative Genomics Viewer (v2.16.2).<br>Splicing analysis of short reads was performed by LeafCutter (v0.2.9) and RegTools (v1.0.0).<br>Long reads were trimmed and mapped by Porechop_ABI (v0.5.0) and Minimap2 (v2.26-r1175), respectively.<br>Transcript structures were predicted based on long reads by StringTie2 (v2.2.1), IsoQuant (v3.3.1), ESPRESSO (v1.3.2), Bambu (v3.4.0), Flair (v0.1), FLAMES (v0.1), TALON (v6.0.1), RNA-Bloom2 (v2.0.1), and GMAP (v2023-07-20).<br>The workflow of transcript-structure identification was implemented as IsoRefiner (v1.0.0).<br>Transcript structures were predicted based on short reads by StringTie2 (v2.2.1), Scallop2 (v1.1.2), Bowtie2 (v2.2.5), TopHat2 (v2.1.1), and Cufflinks (v2.2.1).<br>Comparison and merging of transcript structures were performed by GffCompare (v0.12.6).<br>Simulation of long and short reads was performed by SQANTI-SIM (v0.2.0).<br>Counting of junction-supporing reads were performed by SQANTI3 (v5.3.5).<br>Open reading frames on transcripts were predicted by TransDecoder (v5.7.1).<br>Cloud computational resources were deployed by ParallelCluster (v3.3.1).<br>Basic statistical and multivariate analyses were performed using pandas (v1.5.1), numpy (v1.20.3), scipy (v1.13.1) and scikit-learn (v1.1.3) in python3.

For manuscripts utilizing custom algorithms or software that are central to the research but not yet described in published literature, software must be made available to editors and reviewers. We strongly encourage code deposition in a community repository (e.g. GitHub). See the Nature Portfolio guidelines for submitting code & software for further information.

Data

Policy information about availability of data

All manuscripts must include a data availability statement. This statement should provide the following information, where applicable:

- Accession codes, unique identifiers, or web links for publicly available datasets
- A description of any restrictions on data availability
- For clinical datasets or third party data, please ensure that the statement adheres to our policy

All of our sequence data have been deposited in the DDBJ/ENA/GenBank Sequence Read Archive under BioProject ID PRJDB19918.
The public datasets are available under accession number GSE126543, GSE121569, and PRJEB42763.
The source data supporting this study are provided in Supplementary Data.
IsoRefiner is available at https://github.com/rkajitani/IsoRefiner.

Research involving human participants, their data, or biological material

Policy information about studies with human participants or human data. See also policy information about sex, gender (identity/presentation), and sexual orientation and race, ethnicity and racism.

Reporting on sex and gender	n/a
Reporting on race, ethnicity, or other socially relevant groupings	n/a
Population characteristics	n/a
Recruitment	n/a
Ethics oversight	n/a

Note that full information on the approval of the study protocol must also be provided in the manuscript.

Field-specific reporting

Please select the one below that is the best fit for your research. If you are not sure, read the appropriate sections before making your selection.

☒ Life sciences ☐ Behavioural & social sciences ☐ Ecological, evolutionary & environmental sciences

For a reference copy of the document with all sections, see nature.com/documents/nr-reporting-summary-flat.pdf

Life sciences study design

All studies must disclose on these points even when the disclosure is negative.

Sample size	No formal statistical power calculation was performed prior to the study. Instead, sample sizes were determined based on a balance between experimental feasibility and the need for sufficient data to support RNA-seq analysis and downstream computational tool development. These

	numbers reflect the feasible number of replicates given available resources, and are consistent with common practice in similar transcriptomic studies.
Data exclusions	No data exclusions were made in this study.
Replication	Short-read RNA-seq experiment for iPSCs and untreated-motor neurons were replicated in 3 and 6 biological replicates, respectively. Short- and long-read RNA-seq experiment for scramble and TDP-43 knockdown in motor neurons were replicated in 4 and 5 biological replicates, respectively.
Randomization	Randomization was not required in this study, as the use of homogeneous cell lines minimized experimental variability and eliminated the need for allocation procedures.
Blinding	Blinding was not employed in this study, as it does not involve a clinical trial setting where participant or assessor expectations could introduce bias. Furthermore, sample annotation was integral to the experimental design and necessary for correct data interpretation, making blinding impractical and unnecessary.

Reporting for specific materials, systems and methods

We require information from authors about some types of materials, experimental systems and methods used in many studies. Here, indicate whether each material, system or method listed is relevant to your study. If you are not sure if a list item applies to your research, read the appropriate section before selecting a response.

Materials & experimental systems

n/a	Involved in the study
☒	☐ Antibodies
☐	☒ Eukaryotic cell lines
☒	☐ Palaeontology and archaeology
☒	☐ Animals and other organisms
☒	☐ Clinical data
☒	☐ Dual use research of concern
☒	☐ Plants

Methods

n/a	Involved in the study
☒	☐ ChIP-seq
☒	☐ Flow cytometry
☒	☐ MRI-based neuroimaging

Eukaryotic cell lines

Policy information about cell lines and Sex and Gender in Research

Cell line source(s)	771-3G iPSCs used in this study were purchased from Reprocell. 771-3G iPSCs were established from endothelial progenitor cells (derived from peripheral blood) from human male.
Authentication	771-3G iPSC lines was validated to have normal male karyotype.
Mycoplasma contamination	The supplier has confirmed that the cells are negative for mycoplasma contamination.
Commonly misidentified lines (See ICLAC register)	No commonly misidentified cell lines were used in this study.

Plants

Seed stocks	Our study did not involve the use of plants.
Novel plant genotypes	Our study did not involve the use of plants.
Authentication	Our study did not involve the use of plants.
