# Supplementary material for: Long-read RNA sequencing unveils a novel cryptic exon in MNAT1 along with its full-length transcript structure in TDP-43 proteinopathy
Source: Commun Biol. 2025 Jul 16;8:1056. doi: 10.1038/s42003-025-08463-4 (PMC12267460; doi:10.1038/s42003-025-08463-4)
Supplement: Supplementary file 1 — Transparent Peer Review file [file 42003_2025_8463_MOESM1_ESM.pdf]

nature portfolio

Peer Review File

Long-read RNA sequencing unveils a novel cryptic exon in *MNAT1* along with its full-length transcript structure in TDP-43 proteinopathy

Corresponding Author: Dr Ryo Kunimoto

This file contains all reviewer reports in order by version, followed by all author rebuttals in order by version.

Version 0:

Reviewer comments:

Reviewer #1

(Remarks to the Author)
In this work, Tanaka et al. developed a workflow, referred to as IsoRefiner, integrating the use of a number of existing packages to analyze combined short-read and long-read RNA sequencing data with the aim of determining the full-length structure of cryptic exon-containing transcripts. The workflow is applied to the detection of the effect of TDP-43 depletion, known to promote cryptic exon splicing, on the structure of the MNAT1 transcript.

Major points:
- A number of transcripts incorporating spliced cryptic exons have been identified previously, most notably STMN2 and UNC13A. The authors detected STMN2 in their analysis, but not UNC13A. An explanation for this should be provided. Is it because TDP-43 down-regulation is not efficient enough in their experimental paradigm or is it due to their data analysis method used?
- After extensive filtering, seven cryptic exons were identified; from these, a cryptic exon in MNAT1 was selected for further analysis; this choice should be justified.
- Cryptic exon inclusion in MNAT1 from patient data is not convincing.
- The authors suggest several possibilities fort the consequence of cryptic exon inclusion on the processing of the MNAT1 transcript, including targeting for nonsense-mediated decay or generation of a truncated MAT1 protein. These possibilities should be tested experimentally.
- The authors indicate in the Discussion that abnormal processing of the MNAT1 transcript could be relevant to the pathobiology of amyotrophic lateral sclerosis and frontotemporal dementia; how this could be the case they should be discussed.

Minor point.
- A simplified version of the full-length transcript structure from Supplementary Figure 6 should be included in the main section of the manuscript.

Reviewer #2

(Remarks to the Author)
In this work, Tanaka et al. present a novel approached called IsoRefiner to identify full length alternatively spliced transcripts from long read sequencing. As a model to assess IsoRefiner's ability to detect novel transcripts Tanaka and colleagues focused on splicing changes, with emphasis on cryptic splicing, in response to the knockdown of TDP-43 in human iPSC-

derived motor neuron cultures. Here they found that upon TDP-43 loss of function they could identify previously characterized and novel cryptic spliced transcripts. In particular, MNAT1 was selected for validation using RT-qPCR and was validated through mining publicly available databases from patients with TDP-43 pathology involving TDP-43 loss of function. This helps to highlight the potential disease relevance of their findings.

Overall, this manuscript adds to the field identifying cryptic splicing events related to TDP-43 loss of function by developing a novel approach to increase sensitivity to uncover new biology. This study contains some elegant sequencing and analysis approaches and logical and well performed experiments to support their findings as it relates to human biology and disease. The findings about MNAT1 are potentially of high interest to the field and the discussion brings up many interesting questions and ideas for future studies. However, as the manuscript is presented, the advances in technology development and how it can uncover novel splicing events that are biologically significant are not fully convincing in part due to the presentation of data and in part due to a few missing experiments and validation. With that said, with some major revisions to the text and potentially a few experiments, this manuscript could be suitable for publication and would be of interest to a general audience, particularly to those studying RNA splicing and TDP-43 associated neurodegeneration.

Major Comments:

The advantages of the IsoRefiner approach were only apparent to me after in depth reading of the results, discussion, methods, and supplementary data, with some additional knowledge of the field. While all the content to make this an exciting new pipeline for the field is present in the study, it takes some work for the reader to appreciate the advantages. The results section should contain as much convincing evidence as possible without the need for further investigation from the readers perspective. Presenting some comparison between short read and long read sequencing results and how IsoRefiner can uncover novel transcripts or provide better accuracy in the main results and figures will help the reader to appreciate why this pipeline is needed for the field. Presenting these as number of DEGs or number of uniquely identified splicing event will support the initial findings. Furthermore, is unclear based on the long read results how IsoRefiner is superior to RNA-Bloom2 specifically as they appear to function approximately equal. While it is encouraging that there is reproducibility between the approaches, comparisons between the two approaches should be made clear. It seems as if the biggest advancement is in the computational processing abilities, but this was not made clear until late in the discussion but a direct comparison in the figures would better highlight this major advantage. Comparisons of differentially spliced transcripts between the two approaches from the same dataset (i.e. venn diagram comparison) may help better highlight the different abilities of the two pipelines and may help to uncover some interesting strengths and limitations.

Increasingly the field is excited about cryptic exons as a readout of TDP-43 function. However, many cryptic missplicing events occur in response to TDP-43 loss of function, but only a few are seemingly meaningful and even fewer seem to be reproducible. As presented in this manuscript it is unclear which category MNAT1 falls into, thus some expansion into the validity and biology of MNAT1 is warrented. The analysis of publicly available datasets nicely demonstrates that this is likely reproducible and related to disease, however further validation would be beneficial. Determining the reproducibility of this exon through patient or in vitro datasets and specificity or generalizability across types/cell lines/model organisms would help to solidify MNAT1 splicing as a potentially important gene for further study.

The frameshift mutation leading to novel sequence and premature stop codon is potentially very interesting suggesting there is a protein level change similar to that of HDGFL2 (Calliari et al 2024). Any indication that this can be observed on the protein level by a shift in molecular weight, protein level change, generation of a functional minigene, or identification of this peptide in patient proteomic datasets would significantly increase the impact of this cryptic splicing event. An AlphaFold3 structural comparison between WT and cryptic spliced MNAT1 would also likely yield interesting results. The potential for novel antibodies against these peptides resulting from the frameshift would be exciting as a simple marker of loss of TDP-43 function. The comparisons to HDGFL2 should be included in the discussion as this would significantly impact the field if it can be validated on the protein level.

Minor comments:

Should be clarified that nuclear to cytoplasmic mislocalization is a key driver of TDP-43 loss of function in disease causing the retention of cryptic exons.

Lines 75 – 79 reference a recall rate of 0.5 and 0.4, however these numbers are not entirely clear to non-computational experts. My assumption is a perfect recall rate is 1, thus 0.5 indicates ~50% recall rate. It should be clarified for more general comprehension.

On a similar note, recall and precision are two important concepts for assessing the performance of RNA-seq approaches to map transcript structures, of which there is often a trade-off between the two. For a general audience there should be a brief mention of this in the introduction.

It would be beneficial to present the relative knockdown of TDP-43 on the protein level as there is high levels of autoregulation and often a major discrepancy between TDP-43 protein and TARDBP transcript expression. This will help to understand the magnitude of TDP-43 loss of function required to observe these splicing changes (i.e. sensitivity of splicing event to TDP-43 loss of function).

The RNA sequencing approach to validate TARDBP knockdown in iPSC-MNs should be made explicitly clear in the results (i.e. short-read RNA seq + analysis pipeline). It was unclear whether short or long read RNA seq was applied until later in

the manuscript and unclear which pipeline was used to validate DEGs and splicing changes without more in depth investigation.

Highlighting PFKP in figure 2 and the text may help support TDP-43 loss of function as this was found alongside STMN2 to be downregulated in TDP-43 knockdown and disease conditions (Klim et al. 2019, Rothstein et al. 2023).

Figure 2B may be better suited as a supplementary figure as it does not present new biology generated from the study but simply validates the iPSC differentiation.

A simple comparison between the short read sequencing dataset in figure 2 and publicly available datasets would help to demonstrate the consistency/reproducibility in this study compared to other similar studies on TDP-43 loss of function. This will further strengthen any novel findings using the new IsoRefiner approach.

Reviewer #3

(Remarks to the Author)
Summary:
This study combined short- and long-read sequencing to investigate TDP-43-dependent splicing events and provide this field with a novel workflow IsoRefiner for analysis. The discussion of the biological significance of the findings, particularly regarding MNAT1 is clear and reasonable. However, the paper would benefit from more explanation of the tool selection process and a Venn diagram to compare the identified genes with those from previous studies. Additionally, further experimental validation and a discussion of future therapeutic implications would strengthen the paper. Overall, the study is methodologically sound and provides valuable insights into the role of cryptic exons in TDP-43 proteinopathy

1. This study is (from my knowledge) the first to utilize long-read sequencing to investigate TDP-43 knockdown (KD) in the context of neurodegenerative diseases. However, the target gene MNAT1, has been previously identified in TDP-43-related pathology (PMID: 35197626, 35197628). It would be beneficial for the readers to include a Venn diagram comparing the genes identified in the current study with those from previous studies using short-read sequencing or other methods. This would help clarify the unique contributions of this work and highlight any overlapping genes that have been consistently associated with TDP-43 dysfunction.

2. The authors developed IsoRefiner, a novel workflow for analyzing long-read RNA-seq data, and compared it with five individual tools (Bambu, ESPRESSO, IsoQuant, StringTie2, and RNA-Bloom2). However, the rationale for selecting these specific tools is not thoroughly discussed. It would be helpful to provide a more detailed explanation of why these tools were chosen over other existing ones (eg. TALON, FLAMES…), especially given the rapidly evolving of long-read RNA-seq analysis tools.

3. While the paper is heavily focused on bioinformatics and methodological advancements, the biological significance of MNAT1 in the context of TDP-43 proteinopathy and neurodegenerative diseases could be further elaborated. The author provide potential scenarios of MNAT1 expression is affected by the inclusion of cryptic exon, however, experimental validation at the protein level in TDP-43 KD models or patient samples might be helpful to understand its potential contribution to ALS/FTD pathology.

Overall, the paper is well-written and presents a robust methodological framework for analyzing long-read RNA-seq data. The integration of short- and long-read sequencing is a strong point, as it addresses the limitations of each technology and provides a more comprehensive view of transcript isoforms. However, the current manuscript would be further strengthened by: 1. providing additional experimental validation of the truncated MNAT1 protein (size change/any mislocolization/function loss, which will impact cellular processes in motor neurons? 2. Cross-checking with previous published work and identified targets. 3. The paper could conclude with a more forward-looking discussion on how the findings could be applied to therapeutic development or further mechanistic studies. For example, could targeting the MNAT1 cryptic exon or its downstream effects be a potential therapeutic strategy for ALS/FTD?

Version 1:

Reviewer comments:

Reviewer #1

(Remarks to the Author)
The authors have addressed the points I made in my initial review.

Reviewer #2

(Remarks to the Author)

Tanaka et al. have substantially improved their manuscript “Long-read RNA sequencing unveils a novel cryptic exon in MNAT1 along with its full-length transcript structure in TDP-43 proteinopathy”. Initially, they presented a novel approached called IsoRefiner to identify full length alternatively spliced transcripts from long read sequencing. They used a knockdown model of TDP-43 loss of function as a proof of concept to identify novel cryptic mis-spliced transcripts and nominated MNAT1 as a potential cryptic mis-splicing event of interest. However, several issues were noted including lack of clarity for how their approach is an improvement over existing approaches, lack of direct comparisons with the literature and available datasets, and overall questions regarding the biological relevance of MNAT1.

This version of the manuscript addressed nearly all of the reviewer’s comments. The changes made in both the description and presentation comparing different pipelines for analysis greatly improved clarity, particularly for non-computational scientists. Importantly, validation of MNAT1 cryptic splicing in iPSC motor neurons and iNeurons, but not neuronal-like cancer cell lines is a biologically interesting finding highlighting reproducibility and specificity for cells implicated in ALS. Overall I believe that this manuscript will be of interest to the long-read sequencing, splicing, TDP-43, and ALS fields.

With that, I have no major comments to be addressed with this version of the manuscript and believe it is appropriate for publication, however there are a few minor comments which may be considered.

Minor Comments:

- While I can agree with the authors statement regarding controversy surrounding mislocalization in in vitro experiments, this touches on a highly nuanced subject reflecting the field’s struggles to fully recapitulate disease conditions in vitro. It is well defined that in disease condition TDP-43 is found in aggregates in the cytoplasm. Work over the past decade has revealed that this is likely the culmination of 2 independent processes that are tightly linked (mislocalization and aggregation) (https://doi.org/10.1186/s13024-020-00397-1). Knockdown models of TDP-43 are useful to mimic loss of function which is presumed to better correlate with disease – particularly with regard to splicing – than gain of functon(https://doi.org/10.1186/s13024-024-00732-w). Indeed, recent work has suggested that loss of function can occur upstream of mislocalization (https://doi.org/10.1007/s00401-024-02705-1) however this is also upstream of conventional cytoplasmic aggregates. The current overall convention is that mislocalization causes loss of function which results in splicing, particularly cryptic mis-splicing. Without these distinctions, the use of a knockdown model is missing rationalization. At a minimum, it should mention somewhere that TDP-43 toxicity is thought to be a result of both loss and gain of function mechanisms, with loss of function being strongly linked with splicing alterations in disease.
- The comparisons observed in Supplemental Figure 3D are quite striking. The authors should consider incorporating into the main figures as it strongly supports the strengths of their approach.
- I agree inferences based on AlphaFold modeling can be speculative without rigorous experimental support. It may still be worth-while speculating in the discussion the implications of potential structural changes which may spark future experimentation and exploration. I.e. no data required, simply discussion as there is clearly a discussion to be had for biochemists in the field surrounding MNAT1 cryptic exons.
- A brief mention for the need of cross-species validation should be included as many cryptic mis-splicing events are human specific bringing forth interesting biological questions but also difficulties for animal modelling of disease. Ideally, a simple alignment would at least indicate whether the exon exists in a model genome (i.e. mice), but that may draw more questions beyond the scope of this study.
- Line 411: “Based on our data, the scenario involving NMD appears more likely” seems completely speculative and irrelevant to the conversation as NMD would functionally lead to a knockdown? I would probably just remove this statement.
- The authors switch between “iPS” and “iPSC” nomenclature even within the same cell types (i.e. “iPS-derived motor neurons” on Line 377 and “iPSC-derived motor neurons” on Line 378)
- Line 93: “long read” is missing a hyphen
- Line 681: “short read” is missing a hyphen
- Line 149: reference or citation of the iPSC motor neuron differentiation approach should be mentioned for clarity in the field as various techniques could yield different results.

Reviewer #3

(Remarks to the Author)
The authors has addressed all my comments. I appreciate the clarifications provided and the additional data added. I recommend acceptance of this manuscript.

Open Access This Peer Review File is licensed under a Creative Commons Attribution 4.0 International License, which permits use, sharing, adaptation, distribution and reproduction in any medium or format, as long as you give appropriate credit to the original author(s) and the source, provide a link to the Creative Commons license, and indicate if changes were made.
In cases where reviewers are anonymous, credit should be given to 'Anonymous Referee' and the source.
The images or other third party material in this Peer Review File are included in the article's Creative Commons license, unless indicated otherwise in a credit line to the material. If material is not included in the article's Creative Commons license and your intended use is not permitted by statutory regulation or exceeds the permitted use, you will need to obtain permission directly from the copyright holder.
To view a copy of this license, visit https://creativecommons.org/licenses/by/4.0/

Dr. Reviewers,

We sincerely thank you for your careful review of our manuscript and for the constructive and insightful comments. We appreciate the opportunity to revise our manuscript and have addressed each of the points raised to the best of our ability.

Below, we provide our point-by-point responses to the reviewers' comments. Reviewers' comments are shown, and our responses are provided in blue text following each comment. In addition to the revised manuscript, we have also prepared a version in which all changes are clearly indicated by yellow highlights in markup manuscript.

Thank you very much for your consideration. We look forward to hearing from you.

Reviewer #1 (Remarks to the Author):

In this work, Tanaka et al. developed a workflow, referred to as IsoRefiner, integrating the use of a number of existing packages to analyze combined short-read and long-read RNA sequencing data with the aim of determining the full-length structure of cryptic exon-containing transcripts. The workflow is applied to the detection of the effect of TDP-43 depletion, known to promote cryptic exon splicing, on the structure of the MNAT1 transcript.

Major points:

- A number of transcripts incorporating spliced cryptic exons have been identified previously, most notably STMN2 and UNC13A. The authors detected STMN2 in their analysis, but not UNC13A. An explanation for this should be provided. Is it because TDP-43 down-regulation is not efficient enough in their experimental paradigm or is it due to their data analysis method used?

Author response: UNC13A was originally found in the tissue derived from cortical brain of ALS-FTLD patients (Ma et al., Nature, 2022, PMID: 35197626) and cortical like iPS-derived neurons (Brown et al., Nature, 2022, PMID: 35197628). Additionally, it has been reported that UNC13A is highly expressed in cortical brain regions but lower in the spinal cord (Brown et al., Nature, 2022, PMID: 35197628). The cells we used in this study are motor neurons rather than cortical neurons. Taking these findings together, it is not surprising that the reported UNC13A cryptic exon was not detected in our motor neurons.

- After extensive filtering, seven cryptic exons were identified; from these, a cryptic exon in MNAT1 was selected for further analysis; this choice should be justified.

Author response: Among the final seven cryptic exon candidates, MNAT1 showed the largest dynamic range in splicing changes (Percent Spliced In, PSI) between scramble and knockdown. In addition, the visualization analysis of read coverages exhibited the sharpest peak

for MNAT1. To support this, we have added a new figure quantifying the level of cryptic exon inclusion and mentioned in the text (Fig. 4B; line 281-283).

- Cryptic exon inclusion in MNAT1 from patient data is not convincing.

Author response: To support the observation of MNAT1 cryptic exon inclusion in the patient dataset, we added new quantitative data, showing significantly higher cryptic exon inclusion in the TDP-43 negative group compared to the TDP-43 positive group. These data have been incorporated into the text (Fig.6D; line 363-366).

- The authors suggest several possibilities fort the consequence of cryptic exon inclusion on the processing of the MNAT1 transcript, including targeting for nonsense-mediated decay or generation of a truncated MAT1 protein. These possibilities should be tested experimentally.

Author response: We fully agree that such validation would strengthen the conclusions of our study. However, the cell culture system employed in this study requires over three months to establish and relies on highly specialized techniques. Unfortunately, due to the departure of Dr. Naohiro Sunamura - a key member of our team with the necessary expertise - we are currently unable to conduct additional experiments involving new cell cultures to investigate changes at the protein level.

We have discussed this limitation with the editorial board and, in light of the current circumstances, we have revised the manuscript by incorporating additional computational analyses and expanding the discussion. Although we were unable to perform experimental verification at the protein level, we have newly added evidence that strengthens the hypothesis that the cryptic exon-containing MNAT1 transcript is a NMD target (line 323-330, line342-349; Fig 5E, 6B).. We believe that these improvements sufficiently address the core concerns raised by the reviewers, and we hope to pursue the suggested experimental validation in future work.

(Same response to the Reviewer #3's comment.)

- The authors indicate in the Discussion that abnormal processing of the MNAT1 transcript could be relevant to the pathobiology of amyotrophic lateral sclerosis and frontotemporal dementia; how this could be the case they should be discussed.

Author response: As suggested by the reviewer #1, we have added the suggested content to manuscript on Discussion (line 410-418).

Minor point.

- A simplified version of the full-length transcript structure from Supplementary Figure 6 should be included in the main section of the manuscript.

Author response: As advised, we have included a schematic model of the full-length structure of MNAT1 transcript in the main figure (Fig. 5A).

Reviewer #2 (Remarks to the Author):

In this work, Tanaka et al. present a novel approached called IsoRefiner to identify full length alternatively spliced transcripts from long read sequencing. As a model to assess IsoRefiner's ability to detect novel transcripts Tanaka and colleagues focused on splicing changes, with emphasis on cryptic splicing, in response to the knockdown of TDP-43 in human iPSC-derived motor neuron cultures. Here they found that upon TDP-43 loss of function they could identify previously characterized and novel cryptic spliced transcripts. In particular, MNAT1 was selected for validation using RT-qPCR and was validated through mining publicly available databases from patients with TDP-43 pathology involving TDP-43 loss of function. This helps to highlight the potential disease relevance of their findings.

Overall, this manuscript adds to the field identifying cryptic splicing events related to TDP-43 loss of function by developing a novel approach to increase sensitivity to uncover new biology. This study contains some elegant sequencing and analysis approaches and logical and well performed experiments to support their findings as it relates to human biology and disease. The findings about MNAT1 are potentially of high interest to the field and the discussion brings up many interesting questions and ideas for future studies. However, as the manuscript is presented, the advances in technology development and how it can uncover novel splicing events that are biologically significant are not fully convincing in part due to the presentation of data and in part due to a few missing experiments and validation. With that said, with some major revisions to the text and potentially a few experiments, this manuscript could be suitable for publication and would be of interest to a general audience, particularly to those studying RNA splicing and TDP-43 associated neurodegeneration.

Major Comments:

The advantages of the IsoRefiner approach were only apparent to me after in depth reading of the results, discussion, methods, and supplementary data, with some additional knowledge of the field. While all the content to make this an exciting new pipeline for the field is present in the study, it takes some work for the reader to appreciate the advantages. The results section should

contain as much convincing evidence as possible without the need for further investigation from the readers perspective. Presenting some comparison between short read and long read sequencing results and how IsoRefiner can uncover novel transcripts or provide better accuracy in the main results and figures will help the reader to appreciate why this pipeline is needed for the field. Presenting these as number of DEGs or number of uniquely identified splicing event will support the initial findings. Furthermore, is unclear based on the long read results how IsoRefiner is superior to RNA-Bloom2 specifically as they appear to function approximately equal. While it is encouraging that there is reproducibility between the approaches, comparisons between the two approaches should be made clear. It seems as if the biggest advancement is in the computational processing abilities, but this was not made clear until late in the discussion but a direct comparison in the figures would better highlight this major advantage. Comparisons of differentially spliced transcripts between the two approaches from the same dataset (i.e. venn diagram comparison) may help better highlight the different abilities of the two pipelines and may help to uncover some interesting strengths and limitations.

Author response: To clarify the advantages of combining short and long reads as well as using IsoRefiner, we have added several new results and explanations to the revised manuscript. By comparing the results from short and long reads in both simulations and the actual data, we have demonstrated that employing long reads and IsoRefiner is suitable for identifying transcript structures (line 137–144 and 273–277; Supplementary Fig. 1 and 7D).

With actual data, RNA-Bloom2 outputs a very large number of transcript structures, many of which appear to be false positives, creating a practical issue; however, this problem was not sufficiently explained in the original manuscript. Therefore, in this revision, we have compared the number of transcripts produced by RNA-Bloom2 and IsoRefiner, and demonstrated RNA-Bloom2's drawbacks through evaluating the accuracy of novel transcript splicing junctions with short reads (line 249–259; Supplementary Fig. 7A).

Furthermore, we examined significant splicing events in the actual data detected by short and long reads and investigated their overlap with a Venn diagram. The results indicated that the short- and long-read results largely differed (line 261–273; Supplementary Fig. 7BC), suggesting that our strategy involving the use of short reads is effective for quantification and for detecting

such significant splicing events. All of the results and discussions above have been added to the main text.

Increasingly the field is excited about cryptic exons as a readout of TDP-43 function. However, many cryptic missplicing events occur in response to TDP-43 loss of function, but only a few are seemingly meaningful and even fewer seem to be reproducible. As presented in this manuscript it is unclear which category MNAT1 falls into, thus some expansion into the validity and biology of MNAT1 is warrented. The analysis of publicly available datasets nicely demonstrates that this is likely reproducible and related to disease, however further validation would be beneficial. Determining the reproducibility of this exon through patient or in vitro datasets and specificity or generalizability across types/cell lines/model organisms would help to solidify MNAT1 splicing as a potentially important gene for further study.

Author response: Following your advice, we confirmed the reproducibility and generality using publicly available datasets. In vitro, we examined the presence of the MNAT1 cryptic exon in four cell types from other groups: iPS-derived motor neurons, iPS-derived cortical neurons (iNeurons), SK-N-BE(2), and SH-SY5Y. Although we did not detect the cryptic exon in the two neuroblastoma cell lines (SK-N-BE(2) and SH-SY5Y), it was detected in both the motor neurons and cortical neurons. In vivo, we also quantitatively identified the cryptic exon in the frontal cortex of ALS-FTD patients, as additional data supplementary to Figure 6. Together, these results strongly support the reproducibility and general relevance of the MNAT1 cryptic exon in neurons. These data have been newly included in the manuscript and figure (line 334-342; Fig. 6A and Fig. 6D, and Supplementary Fig. 9A).

The frameshift mutation leading to novel sequence and premature stop codon is potentially very interesting suggesting there is a protein level change similar to that of HDGFL2 (Calliari et al 2024). Any indication that this can be observed on the protein level by a shift in molecular weight, protein level change, generation of a functional minigene, or identification of this peptide in patient proteomic datasets would significantly increase the impact of this cryptic splicing

event. An AlphaFold3 structural comparison between WT and cryptic spliced MNAT1 would also likely yield interesting results. The potential for novel antibodies against these peptides resulting from the frameshift would be exciting as a simple marker of loss of TDP-43 function. The comparisons to HDGFL2 should be included in the discussion as this would significantly impact the field if it can be validated on the protein level.

Author response: As you have pointed out, both MNAT1 and HDGFL2 exhibit similar phenomena as readouts of TDP-43 loss of function. However, according to previous reports, HDGFL2 retains the reading frame even after the inclusion of a cryptic exon, suggesting the possibility of producing a functional protein. In contrast, our newly incorporated analyses in this revision strongly support that cryptic splicing of MNAT1 induces a frameshift, making the transcript likely a target for nonsense-mediated mRNA decay (NMD). Given these fundamental differences in the fate of the transcripts and proteins resulting from the splicing abnormalities, we believe that a direct comparison of these two genes on the same basis would not be appropriate.

Although our newly incorporated analyses in this revision strongly support that MNAT1 becomes a target for NMD, we cannot completely exclude the possibility that a functionally altered protein may still be produced. As shown in Fig. 5D and Supplementary Fig. 8D, the structural changes between the wild-type and cryptically spliced MNAT1 are caused by a frameshift that alters the amino acid sequence of a specific motif, leading to significant structural differences from a structural biology perspective. To further investigate this, we performed structural prediction of the complex containing MNAT1 using Boltz-1, a replication of AlphaFold3. The results are shown below: the left panel displays the structure of wild-type MNAT1, and the right panel shows the structure of cryptic spliced MNAT1. The wild-type structure exhibits an interaction form similar to the previously reported structure (PMID: 32855301), while the cryptic spliced MNAT1 shows a structurally unreasonable and distinct interaction pattern. These findings also further support the loss-of-function hypothesis from a structural perspective. However, as these complex structures are based solely on computational predictions, we would like to include these results only as supplementary information in the response letter rather than in the main manuscript.

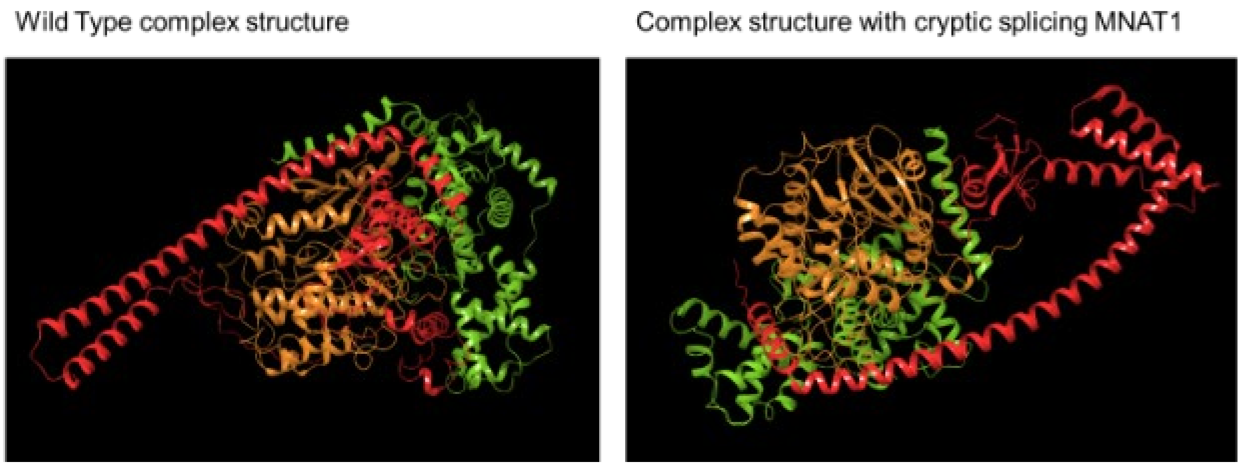

Fig. Left panel: Predicted complex structure containing wild-type MNAT1. Right panel: Predicted complex structure containing cryptically spliced MNAT1. In both panels, MNAT1, CDK7, and CCNH are shown in red, orange, and green, respectively.

Minor comments:

Should be clarified that nuclear to cytoplasmic mislocalization is a key driver of TDP-43 loss of function in disease causing the retention of cryptic exons.

Author response: We respectfully disagree. The translocation of TDP-43 from the nucleus to cytoplasm has been a subject of controversy in *in vitro* experiments (Bilican et al., Proc. Natl. Acad. Sci., 2012, PMID: 22451909; Klim et al., Nat Neurosci., 2019, PMID: 30643292). Therefore, it cannot be considered a definitive phenomenon in iPSC-derived motor neuron.

Lines 75 – 79 reference a recall rate of 0.5 and 0.4, however these numbers are not entirely clear to non-computational experts. My assumption is a perfect recall rate is 1, thus 0.5 indicates ~50% recall rate. It should be clarified for more general comprehension.

Author response: We have added a detailed explanation to the main text (line 79–82).

On a similar note, recall and precision are two important concepts for assessing the performance of RNA-seq approaches to map transcript structures, of which there is often a trade-off between the two. For a general audience there should be a brief mention of this in the introduction.

Author response: Also, we have added a detailed explanation to the main text (line 85–89).

It would be beneficial to present the relative knockdown of TDP-43 on the protein level as there is high levels of autoregulation and often a major discrepancy between TDP-43 protein and TARDBP transcript expression. This will help to understand the magnitude of TDP-43 loss of function required to observe these splicing changes (i.e. sensitivity of splicing event to TDP-43 loss of function).

Author response: We agree that the autoregulation mechanism of TDP-43 is a critical aspect to consider. As the reviewer noted, TDP-43 has an autoregulation mechanism, which makes knockdown experiments time-sensitive. Therefore, we guided our analysis using the expression levels of a more sensitive downstream gene, STMN2, to confirm the loss of TDP-43 function (line 166-168).

The RNA sequencing approach to validate TARDBP knockdown in iPSC-MNs should be made explicitly clear in the results (i.e. short-read RNA seq + analysis pipeline). It was unclear whether short or long read RNA seq was applied until later in the manuscript and unclear which pipeline was used to validate DEGs and splicing changes without more in depth investigation.

Author response: In our workflow, we first performed short-read RNA sequencing to predict the presence of cryptic exons. We then conducted long-read RNA sequencing to investigate the full-length structure of transcripts containing these predicted cryptic exons. Because relying solely on long-read sequencing raises concerns about throughput and read coverage, we chose a strategy in which short-read sequencing guides the identification of cryptic exon-containing transcripts. Since our figure may have caused confusion for these steps, we have

revised Fig. 2A to reflect the points you raised for guiding the correct steps (Fig. 2A) and clarified the text accordingly (line 21-22; line 160).

Highlighting PFKP in figure 2 and the text may help support TDP-43 loss of function as this was found alongside STMN2 to be downregulated in TDP-43 knockdown and disease conditions (Klim et al. 2019, Rothstein et al. 2023).

Author response: We agree with your comment. Under TDP-43 KD condition, the PFKP mRNA expression was decreased. We have newly included this data as evidence supporting TDP-43 loss of function (Fig. 2C; line 166-167).

Figure 2B may be better suited as a supplementary figure as it does not present new biology generated from the study but simply validates the iPSC differentiation.

Author response: We have moved this figure to the supplementary figures (Supplementary Fig. 2C).

A simple comparison between the short read sequencing dataset in figure 2 and publicly available datasets would help to demonstrate the consistency/reproducibility in this study compared to other similar studies on TDP-43 loss of function. This will further strengthen any novel findings using the new IsoRefiner approach.

Author response: To compare our study with previous work, we performed a cross-reference analysis using publicly available datasets. Since we identified the MNAT1 cryptic exon from in vitro samples, we selected in vitro RNA-seq datasets (motor neurons and cortical neurons) from previous studies and applied identical thresholds in LeafCutter for a fair comparison. Upon examining the overlap of genes harboring differential splicing events, MNAT1 was found to overlap between our motor neurons and cortical neurons (Supplementary Fig. 3D). Although MNAT1 was seemingly undetected in another group’s motor neuron dataset,

we suspect this is due to tool sensitivity issues. In fact, quantification analysis of the cryptic exon suggests significant MNAT1 cryptic exon inclusion even in the public motor neuron dataset (Fig. 6A). We also noticed that MNAT1 was listed in the study of Ma et al. Nature, 2022 (PMID: 35197626); however, that figure merely lists splicing events characterized as differentially spliced, without designating them specifically as cryptic exons. Taken together, our additional findings support the reproducibility and general relevance of the MNAT1 cryptic exon in neurons. We have added figures and modified texts to reflect this (Supplementary Fig. 3D, Fig. 6A; line 170-176, line 334-342).

(Same response to the Reviewer #3's comment.)

Reviewer #3 (Remarks to the Author):

Summary:

This study combined short- and long-read sequencing to investigate TDP-43-dependent splicing events and provide this field with a novel workflow IsoRefiner for analysis. The discussion of the biological significance of the findings, particularly regarding MNAT1 is clear and reasonable. However, the paper would benefit from more explanation of the tool selection process and a Venn diagram to compare the identified genes with those from previous studies. Additionally, further experimental validation and a discussion of future therapeutic implications would strengthen the paper. Overall, the study is methodologically sound and provides valuable insights into the role of cryptic exons in TDP-43 proteinopathy

1. This study is (from my knowledge) the first to utilize long-read sequencing to investigate TDP-43 knockdown (KD) in the context of neurodegenerative diseases. However, the target gene MNAT1, has been previously identified in TDP-43-related pathology (PMID: 35197626, 35197628). It would be beneficial for the readers to include a Venn diagram comparing the genes identified in the current study with those from previous studies using short-read sequencing or other methods. This would help clarify the unique contributions of this work and highlight any overlapping genes that have been consistently associated with TDP-43 dysfunction.

Author response: To compare our study with previous work, we performed a cross-reference analysis using publicly available datasets. Since we identified the MNAT1 cryptic exon from in vitro samples, we selected in vitro RNA-seq datasets (motor neurons and cortical neurons) from previous studies and applied identical thresholds in LeafCutter for a fair comparison. Upon examining the overlap of genes harboring differential splicing events, MNAT1 was found to overlap between our motor neurons and cortical neurons (Supplementary Fig. 3D). Although MNAT1 was seemingly undetected in another group's motor neuron dataset, we suspect this is due to tool sensitivity issues. In fact, quantification analysis of the cryptic exon suggests significant MNAT1 cryptic exon inclusion even in the public motor neuron dataset (Fig. 6A). We also noticed that MNAT1 was listed in the study of Ma et al. Nature, 2022 (PMID:

35197626); however, that figure merely lists splicing events characterized as differentially spliced, without designating them specifically as cryptic exons. Taken together, our additional findings support the reproducibility and general relevance of the MNAT1 cryptic exon in neurons. We have added figures and modified texts to reflect this (Supplementary Fig. 3D, Fig. 6A; line 170-176, line 334-342).

(Same response to the Reviewer #2's comment)

2. The authors developed IsoRefiner, a novel workflow for analyzing long-read RNA-seq data, and compared it with five individual tools (Bambu, ESPRESSO, IsoQuant, StringTie2, and RNA-Bloom2). However, the rationale for selecting these specific tools is not thoroughly discussed. It would be helpful to provide a more detailed explanation of why these tools were chosen over other existing ones (eg. TALON, FLAMES…), especially given the rapidly evolving of long-read RNA-seq analysis tools.

Author response: In line with the reviewer #3's comments, we have added an explanation of our rationale for selecting the tools that IsoRefiner uses internally. We also newly tested TALON and FLAMES, confirming their poor performance in simulation-based evaluations (Fig. 1A). Our tool selection was based on simulation performance assessments and computational costs (the number of tools). We observed that Flair, TALON, and FLAMES all had low F1 scores, and in particular, Flair's low precision was deemed to negatively impact the merged results. Although Bambu also had a low F1 score, it achieved the highest precision among the tools tested, so we decided to include it. These explanations have been added to the main text (line 124–129).

3. While the paper is heavily focused on bioinformatics and methodological advancements, the biological significance of MNAT1 in the context of TDP-43 proteinopathy and neurodegenerative diseases could be further elaborated. The author provide potential scenarios of MNAT1 expression is affected by the inclusion of cryptic exon, however, experimental

validation at the protein level in TDP-43 KD models or patient samples might be helpful to understand its potential contribution to ALS/FTD pathology.

Author response: We further explored the generality of the MNAT1 cryptic exon. In vitro, we validated its presence using multiple publicly available RNA-seq datasets beyond our own experimental system. Consequently, we observed MNAT1 CE in iPS-derived motor neurons established by other groups, in SH-SY5Y cell cultures, and in iPS-derived cortical-like neurons (Fig. 6A, Supplementary Fig. 9A). Moreover, to provide more objective evidence for the patient data previously shown in Figure 6, we quantified cryptic exon inclusion, demonstrating that the MNAT1 cryptic exon was significantly increased in TDP-43–negative nuclei (Fig. 6D). We have reflected these additional findings in the figures and the text (Fig. 6A, Supplementary Fig. 9A Fig. 6D; line 334-342, line 363-370).

Overall, the paper is well-written and presents a robust methodological framework for analyzing long-read RNA-seq data. The integration of short- and long-read sequencing is a strong point, as it addresses the limitations of each technology and provides a more comprehensive view of transcript isoforms. However, the current manuscript would be further strengthened by: 1. providing additional experimental validation of the truncated MNAT1 protein (size change/any mislocolization/function loss, which will impact cellular processes in motor neurons?

Author response: As suggested by the reviewer #3, we have added the suggested content to manuscript on Discussion (line 410-418).

We fully agree that such validation would strengthen the conclusions of our study. However, the cell culture system employed in this study requires over three months to establish and relies on highly specialized techniques. Unfortunately, due to the departure of Dr. Naohiro Sunamura - a key member of our team with the necessary expertise - we are currently unable to conduct additional experiments involving new cell cultures to investigate changes at the protein level.

We have discussed this limitation with the editorial board and, in light of the current circumstances, we have revised the manuscript by incorporating additional computational

analyses and expanding the discussion. Although we were unable to perform experimental verification at the protein level, we have newly added evidence that strengthens the hypothesis that the cryptic exon-containing MNAT1 transcript is a NMD target (line 323-330, line342-349; Fig 5E, 6B) We believe that these improvements sufficiently address the core concerns raised by the reviewers, and we hope to pursue the suggested experimental validation in future work.

(Same response to the Reviewer #1 comments.)

2. Cross-checking with previous published work and identified targets.

Author response: By employing external datasets, we performed (1) a gene-level analysis of splicing events (Supplementary Fig. 3D), and (2) a quantitative analysis of MNAT1 cryptic exon inclusion levels (Fig. 6A). These analyses confirmed the presence of the MNAT1 cryptic exon in iPS-derived motor neurons, iPS-derived cortical neurons, and frontal cortex tissue from ALS-FTD patients. We have included these data in the figures and reflected them in the texts (Supplementary Fig. 3D and Fig. 6A; line 170-176, line 334-342, line).

3. The paper could conclude with a more forward-looking discussion on how the findings could be applied to therapeutic development or further mechanistic studies. For example, could targeting the MNAT1 cryptic exon or its downstream effects be a potential therapeutic strategy for ALS/FTD?

Author response: Although our study provides strong evidence that MNAT1 is among the genes regulated by TDP-43 in neurons, it remains unclear whether MNAT1 could serve as a therapeutic target. At this stage, the MNAT1 cryptic exon could be a promising candidate for diagnostic or biomarker use. We believe further investigations are needed to establish MNAT1's therapeutic potential. As demonstrated in the STMN2 papers (PMID: 30643298, 30643292, 36927019), therapeutic applications would require evidence that restoring wild-type MNAT1 protein can rescue phenotypes such as axonal growth. We have reflected this in the texts (line 418-428).

Dear Reviewers,

We sincerely appreciate your careful review of our manuscript and your constructive and insightful comments. We have already addressed the points raised by Reviewers #1 and #3 in the first revision, and in the second revision, we have addressed the additional points raised by Reviewer #2 to the extent possible.

Below, we provide our point-by-point responses to the reviewers' comments. Reviewers' comments are shown, and our responses are provided in blue text following each comment. In addition to the revised manuscript, we have also prepared a version in which all changes are clearly indicated by yellow highlights.

Thank you very much for your consideration. We look forward to hearing from you.

Reviewer #2 (Remarks to the Author):

Minor Comments:

- While I can agree with the authors statement regarding controversy surrounding mislocalization in in vitro experiments, this touches on a highly nuanced subject reflecting the field's struggles to fully recapitulate disease conditions in vitro. It is well defined that in disease condition TDP-43 is found in aggregates in the cytoplasm. Work over the past decade has revealed that this is likely the culmination of 2 independent processes that are tightly linked (mislocalization and aggregation) (https://doi.org/10.1186/s13024-020-00397-1). Knockdown models of TDP-43 are useful to mimic loss of function which is presumed to better correlate with disease – particularly with regard to splicing – than gain of functon(https://doi.org/10.1186/s13024-024-00732-w). Indeed, recent work has suggested that loss of function can occur upstream of mislocalization (https://doi.org/10.1007/s00401-

024-02705-1) however this is also upstream of conventional cytoplasmic aggregates. The current overall convention is that mislocalization causes loss of function which results in splicing, particularly cryptic mis-splicing. Without these distinctions, the use of a knockdown model is missing rationalization. At a minimum, it should mention somewhere that TDP-43 toxicity is thought to be a result of both loss and gain of function mechanisms, with loss of function being strongly linked with splicing alterations in disease.

Author response: We appreciate your comments on the aspect of TDP-43 gain of function. Following your comments, we mentioned the gain of function in the text. (Lines: 146-148)

- The comparisons observed in Supplemental Figure 3D are quite striking. The authors should consider incorporating into the main figures as it strongly supports the strengths of their approach.

Author response: We agree that the importance of Supplemental Figure 3D in the context of comparison with previous studies. However, we would like to emphasize that the full-length transcript structure of *MNAT1* was identified in this study through a novel approach using independently generated data. Accordingly, we consider Supplemental Figure 3D to serve as a supplementary figure rather than a central element of the study.

- I agree inferences based on AlphaFold modeling can be speculative without rigorous experimental support. It may still be worth-while speculating in the discussion the implications of potential structural changes which may spark future experimentation and exploration. I.e. no data required, simply discussion as there is clearly a discussion to be had for biochemists in the field surrounding MNAT1 cryptic exons.

Author response: We add some texts regarding to the protein structural prediction. (Lines: 411-416).

- A brief mention for the need of cross-species validation should be included as many cryptic mis-splicing events are human specific bringing forth interesting biological questions but also difficulties for animal modelling of disease. Ideally, a simple alignment would at least indicate whether the exon exists in a model genome (i.e. mice), but that may draw more questions beyond the scope of this study.

Author response: We fully acknowledge the scientific importance of investigating interspecies homology. However, as this study is consistently focused on human biology, any implications regarding animal models fall outside the scope of the present work and therefore cannot be addressed.

- Line 411: "Based on our data, the scenario involving NMD appears more likely" seems completely speculative and irrelevant to the conversation as NMD would functionally lead to a knockdown? I would probably just remove this statement.

Author response: We removed this sentence from the text.

- The authors switch between "iPS" and "iPSC" nomenclature even within the same cell types (i.e. "iPS-derived motor neurons" on Line 377 and "iPSC-derived motor neurons" on Line 378)

Author response: We standardized the terminology in iPSC.

- Line 93: "long read" is missing a hyphen

Author response: This has been amended.

- Line 681: “short read” is missing a hyphen

Author response: This has been amended.

- Line 149: reference or citation of the iPSC motor neuron differentiation approach should be mentioned for clarity in the field as various techniques could yield different results.

Author response: We cited the reference paper (line: 44).
